# Mapping the “What” and “Where” Visual Cortices and Their Atrophy in Alzheimer's Disease: Combined Activation Likelihood Estimation with Voxel-Based Morphometry

**DOI:** 10.3389/fnhum.2016.00333

**Published:** 2016-06-29

**Authors:** Yanjia Deng, Lin Shi, Yi Lei, Peipeng Liang, Kuncheng Li, Winnie C. W. Chu, Defeng Wang, for the Alzheimer's Disease Neuroimaging Initiative

**Affiliations:** ^1^Department of Imaging and Interventional Radiology, The Chinese University of Hong KongHong Kong, China; ^2^Department of Medicine and Therapeutics, The Chinese University of Hong KongHong Kong, China; ^3^Chow Yuk Ho Center of Innovative Technology for Medicine, The Chinese University of Hong KongHong Kong, China; ^4^Department of Radiology, The Second People's Hospital of ShenzhenShenzhen, China; ^5^Department of Radiology, Xuanwu Hospital, Capital Medical UniversityBeijing, China; ^6^Shenzhen Research Institute, The Chinese University of Hong KongShenzhen, China

**Keywords:** visual perception, activation likelihood estimation, functional magnetic resonance imaging, Alzheimer's disease, voxel-based morphometry

## Abstract

The human cortical regions for processing high-level visual (HLV) functions of different categories remain ambiguous, especially in terms of their conjunctions and specifications. Moreover, the neurobiology of declined HLV functions in patients with Alzheimer's disease (AD) has not been fully investigated. This study provides a functionally sorted overview of HLV cortices for processing “what” and “where” visual perceptions and it investigates their atrophy in AD and MCI patients. Based upon activation likelihood estimation (ALE), brain regions responsible for processing five categories of visual perceptions included in “what” and “where” visions (i.e., object, face, word, motion, and spatial visions) were analyzed, and subsequent contrast analyses were performed to show regions with conjunctive and specific activations for processing these visual functions. Next, based on the resulting ALE maps, the atrophy of HLV cortices in AD and MCI patients was evaluated using voxel-based morphometry. Our ALE results showed brain regions for processing visual perception across the five categories, as well as areas of conjunction and specification. Our comparisons of gray matter (GM) volume demonstrated atrophy of three “where” visual cortices in late MCI group and extensive atrophy of HLV cortices (25 regions in both “what” and “where” visual cortices) in AD group. In addition, the GM volume of atrophied visual cortices in AD and MCI subjects was found to be correlated to the deterioration of overall cognitive status and to the cognitive performances related to memory, execution, and object recognition functions. In summary, these findings may add to our understanding of HLV network organization and of the evolution of visual perceptual dysfunction in AD as the disease progresses.

## Introduction

The human visual cortex is primarily located in, but not confined to, the occipital lobe. It extends into the temporal and parietal lobes and shows complicated cortical distribution. Understanding of the human visual cortex has grown with the development of functional magnetic resonance imaging (fMRI). To date, over a dozen cortical areas have been identified to be involved in visual functions (Sereno et al., 1995; Tyler et al., 2005; Wandell et al., 2005), and the pathway model was introduced to divide these functional areas into dorsal and ventral processing streams (Haxby et al., 1991; Pihlajamaki et al., 2005).

The dorsal pathway, also known as the “where” stream, is an occipito-parietal network which lies between the early visual cortex and those specialized cortical structures involved in the processing of spatial and motion information (Kravitz et al., 2013). The ventral pathway, also known as the “what” stream, is an occipito-temporal network that bridges the early visual cortex and is involved in processing visual identity and feature information (e.g., faces, object identities, colors, and words; Kravitz et al., 2013). To date, a growing number of studies have investigated the anatomical distributions of the ventral and dorsal streams as well as their specializations and conjunctions in the processing of visual perceptions for the different categories (Grill-Spector et al., 2004; Downing et al., 2006; Cichy et al., 2011; Mano et al., 2013). For example, the motion-selective area of V5 (hMT+; Morrone et al., 2000; Huk et al., 2002), the spatial specific brain region of V3a (Tootell et al., 1997; Backus et al., 2001), as well as areas for words (Liu et al., 2008) and faces (Grill-Spector et al., 2004), are anatomically distinct in fMRI.

In recent years, emerging studies have begun to investigate the neural basis of disrupted high-level visual (HLV) functions in Alzheimer's disease (AD). Neuropsychological and neuroimaging findings have demonstrated that both the dorsal (Kavcic et al., 2011; Mandal et al., 2012) and ventral (Kurylo et al., 1996; Adlington et al., 2009) visual streams are impaired during the progression of AD (Delbeuck et al., 2003; Mandal et al., 2012). Because successfully exercising cognitive functions such as memory, executive function and motion perception usually depends upon intact visual perceptual function (Reisberg, 2010; Culham, 2015), it is reasonable to suspect that declines in HLV functions may also contribute to poor cognition in AD. Several studies have demonstrated that deficits in visual perception significantly correlate with poor performance in instrumental activities of daily living (Eslinger et al., 1985), as well as with impaired general cognitive ability (Silveri and Leggio, 1996; Rizzo et al., 2000). This demonstrates the importance of investigating alterations of HLV cortices in AD.

However, due to several sources of study limitations, there are relatively few widely accepted models of alterations of HLV cortices during AD-related pathological progression. First, unlike with primary visual cortices, the “what” and “where” visual streams include the emergence of a series of categories, and processing these types of visions may involve different cortical regions. Because a single fMRI study cannot include a wide variety of paradigms corresponding to different visual categories, it is difficult to obtain a comprehensive overview for the cortical locations of the dorsal and ventral streams. In addition, the issue of crosstalk between the dorsal and ventral streams has been discussed extensively in recent years (Schenk and McIntosh, 2010). Thus, there exist possible regions of conjunction in stream process, and overlaps may even complicate the cortical mapping of “what” and “where” visual functions in intact individuals. Second, due to the variance in methods and relevant parameters used in prior fMRI studies, the anatomical locations and boundaries of the functional areas of the dorsal and ventral streams remain ambiguous. In other words, it remains unclear whether different functional regions across different fMRI studies reflect a true difference in regions of neural activations across different categories of visual targets or are caused by the methodological differences among the studies (e.g., methods of data preprocessing). Overall, due to the lack of comprehensive, systematic, and accurate cortical localizations for HLV cortices, their alterations in AD-related pathological progression have been less than fully investigated to date.

Therefore, this study aims to provide a functionally sorted overview of the HLV cortices of the “what” and “where” visions, and, on this basis, comprehensively investigate the atrophy of these HLV cortices in AD and mild cognitive impairment (MCI). First, the HLV cortices responsible for processing different categories of visual perception were mapped by performing activation likelihood estimation (ALE) on previous relevant fMRI studies. This step was used to provide a functionally sorted overview of the HLV network of the “what” and “where” visions. ALE is a coordinate-based analysis approach for neuroimaging data, which is based upon calculating the overlap between reported foci in different studies, modeling them as probability distributions centered at given coordinates. Therefore, the created ALE map can then be used to indicate inter-study regions of consistent brain activation during a cognitive process which would not be obvious upon initial inspection (Turkeltaub et al., 2002). Second, based upon the subsequent contrast analyses, the resulting specific and conjunctive brain regions responsible for processing different categories were further investigated. It was thus possible to shed light on how interactions among the different categories of visual processing were organized within the brain. For example, “Which brain areas were constantly activated for both ‘what’ and ‘where’ visual stimuli?”. Third, gray matter (GM) volume of these HLV cortices (mapped in previous steps) was measured using voxel-based morphometry (VBM) method, and compared among AD, MCI, and normal control participants. Then, the GM volume of visual cortices identified with atrophy was further correlated to the cognitive scoring. This was to provide a dynamic observation of alterations to the HLV cortices throughout AD progression, as well as their relation to the cognitive deterioration. Therefore, these findings may help to better elucidate the cortical organization of the HLV cortices, as well as the evolution of visual perceptual dysfunction during the progression of AD.

## Materials and methods

### Mapping the cortices of “what” and “where” visions using ALE

#### Literature selection and exclusion criteria

We searched for studies that investigated HLV functions in the PubMed database (http://www.ncbi.nlm.nih.gov/pubmed) using the search terms: “((((((((((‘Visual Perception’[Mesh]) OR ‘Color Perception’[Mesh]) OR ‘Motion Perception’[Mesh]) OR ‘Pattern Recognition, Visual’[Mesh]) OR ‘Perceptual Closure’[Mesh]) OR ‘Perceptual Masking’[Mesh]) OR ‘Space Perception’[Mesh]) OR ‘Depth Perception’[Mesh]) OR ‘Form Perception’[Mesh]) OR ‘Size Perception’[Mesh]) AND ‘Magnetic Resonance Imaging’[Mesh]”. As of April 2016, this search revealed 3430 published, peer-reviewed papers. The inclusion and exclusion criteria for our analyses were as follows:

1. Papers should be published in English, and studies must explicitly refer to the visual cortex as involved in the processing of visual stimuli. Subjects should be healthy adults. Those papers that did not meet the first two criteria were excluded. Only 1558 of the 3430 papers met these criteria.
2. Studies that used fMRI were included. Those only employing other techniques, e.g., positron emission tomography (PET), single-photon emission tomography (SPECT), magnetoencephalography (MEG), transcranial magnetic stimulation (TMS), behavioral measures, and review articles, were excluded. The meta-analysis was limited to fMRI studies because of their comparability in the spatial and temporal resolutions for the ALE analyses. Only 1401 of the remaining 1558 papers met this criterion.
3. Studies that did not image the whole brain (i.e., from the top of the brain to the cerebellum) or did not report the coordinates of the activation clusters were excluded. Any studies using only a region of interest (ROI) analytic method instead of whole-brain analyses were excluded. Only 689 of the remaining 1401 papers survived.
4. Studies that did not report coordinates in the normalized stereotaxic reference systems of either Talairach and Tournoux (1988) or the MNI or that reported coordinates that were not in the Cartesian (XYZ) format were excluded. Seven of the remaining 689 papers were excluded.
5. Studies in which the authors did not attribute their fMRI results directly to a particular visual function or task, or which involved more than one visual function (e.g., color and depth visions) simultaneously, were excluded. Studies using low-level contrasts (target vision vs. baseline, noise or scrambled meaningless images) were included to maintain the homogeneity of the meta-analysis. Studies that only provided the coordinates for comparison with high-level contrasts were excluded. Ultimately, 93 papers fulfilled all of these criteria.

#### Data analysis

The 93 studies fitting within the established criteria were first divided into two major categories of “what” and “where” visions. These included studies of face, object, alphabetic word/letter, logographic word/symbol, scene, body, motion, and spatial visions, respectively. These studies were further classified into eight basic visual categories of motion, space, object, face, alphabetic word/letter (word for short), logographic word/symbol, scene, and body visions (Supplementary Table 1). To ensure the power of the results, visual functions with less than 10 experiments (i.e., body, scene and logographic word visions) were not used to establish an independent category. Consequently, five categories remained.

To identify the specific brain regions responsible for particular visual functions, we subsequently conducted a contrast analysis of each pair from the task categories of face-word, face-object, object-word and “what”–“where” vision. Contrast analyses between motion and spatial vision were not performed due to the large difference in the number of included studies between these two categories, which might have impacted the power of the statistical results.

The Ginger ALE software program, version 2.0 (available at http://www.brainmap.org/ale) with revised ALE algorithm implemented in was used. ALE is a coordinate-based meta-analysis tool that treats significant foci reported in neuroimaging studies not as single points but as spatial probability distributions centered at the given coordinates. ALE maps are then produced by computing the union of activation probabilities for each voxel (Eickhoff et al., 2012; Turkeltaub et al., 2012). First, coordinates of the original studies reported in the Talairach brain map were transformed into the MNI space using the Lancaster transform (Lancaster et al., 2007). The foci from each individual study were smoothed by a full width half maximum (FWHM) value scaled by the study's sample size to model the uncertainty in the spatial location of the activations (Eickhoff et al., 2009). An analytical method was used to determine the null distribution of the ALE statistic, and correction for multiple comparisons was then applied using the false discovery rate (FDR pN; Laird et al., 2005), with *p* < 0.01. A minimum cluster volume of 200 mm^3^ was applied. The statistical level for contrast analysis was also set at *p* < 0.01 (FDR pN corrected) with a minimum volume ≥ 200 mm^3^. The generated ALE maps were overlaid on a Colin_tlrc_2 × 2 × 2 template using the MANGO software program for visualization (http://rii.uthscsa.edu/mango/).

### VBM analysis of HLV cortices in AD and MCI patients

#### Subjects

All of the imaging and neuropsychological test data were taken from the Alzheimer's Disease Neuroimaging Initiative (ADNI) database (adni.loni.usc.edu). For more information about the ADNI dataset, as well as its subject inclusion and exclusion criteria, please see the ADNI dataset for more details in the Supplementary Material and the ADNI website at http://adni.loni.usc.edu/methods/documents/. For the current study, subjects with glaucoma, congenital blindness, or excessive head motion during MRI scanning were additionally excluded due to the nature of this study. Moreover, to ensure comparability between structural and functional results in future studies, only subjects with both three-dimensional T1-weighted imaging (3D T1WI) and resting-state functional MRI scans were included. Finally, 44 CN (normal control), 52 EMCI (early MCI), 35 LMCI (late MCI), and 30 AD subjects were included.

#### Image acquisition and data preprocessing

All of the subjects were scanned using 3T Philips MRI scanners. The high resolution 3D T1WI were acquired using magnetization-prepared rapid acquisition gradient-echo (MPRAGE) with TR = 6.8 ms, TE = 3.1 ms, 170 slices, FOV = 256 mm, matrix size = 256 × 256, and slice thickness = 1.2 mm.

All 3D T1WI data were processed using the VBM8 toolbox (http://dbm.neuro.uni-jena.de/vbm) implemented in the SPM8 software (http://www.fil.ion.ucl.ac.uk/spm/). Brain images were linearly registered (12-parameter affine) to approximate the MNI space and segmented into GM, white matter, and cerebrospinal fluid images. The GM images were further normalized using DARTEL (Diffeomorphic Anatomical Registration using Exponentiated Lie algebra; Ashburner, 2007), Jacobian modulated and smoothed using an 8 mm FWHM Gaussian kernel. Finally, the mean GM volume was extracted for each visual ROI. Here, all visual ROIs were made by using the clusters obtained from the ALE results in part *3.1*, corresponding to the “face,” “object,” “word,” “motion,” and “spatial” visual regions. In total, the GM volume of 36 ROIs (11 for face vision, 7 for object vision, 5 for word vision, 7 for motion vision, and 6 for spatial vision, see **Table 3**) was calculated for each subject in the CN, EMCI, LMCI, and AD groups.

#### Statistical analyses

The demographics and mini-mental state examination (MMSE) scores of the subjects were compared among the CN, EMCI, LMCI, and AD groups by one-way ANOVA (or Kruskal–Wallis *H* test if the data were not normally distributed), with a statistical significance level at *p* < 0.05. For the evaluation of GM volume difference among four groups, a multivariate analysis of variance (MANCOVA) was firstly conducted to evaluate the main effect of group. Then, analysis of covariance (ANCOVA) was performed respectively for each ROI to compare the GM volume among four groups. The whole-brain volume was added as a covariate in both the MANCOVA and ANCOVA analyses to ensure that observed GM volume differences in HLV cortices among groups were independent of the variance in whole-brain volume across subjects. A statistical level of 0.0014 was used to correct for 36 times of comparisons for all the ROIs (i.e., Bonferroni method, 0.05/36 ≈ 0.0014), and further *Post-hoc* analyses between each pair of groups were also corrected with the Bonferroni correction. In addition, the GM volume of each ROI identified with significant atrophy in ADs or MCIs (finally turned out to be 25 ROIs totally in our results) was correlated to the scores of clock drawing test, category fluency-animal, trail making test A & B, logical memory test I & II, Boston naming test, MMSE, and whole-brain mean GM volume using Pearson correlation (or Spearmen correlation if the data were not normally distributed). A statistical level of 0.002 was used to correct for multiple times of correlations (i.e., Bonferroni method, 0.05/25 = 0.002).

## Results

### Cortices for “what” and “where” visions acquired from ALE

**Figure 1:**
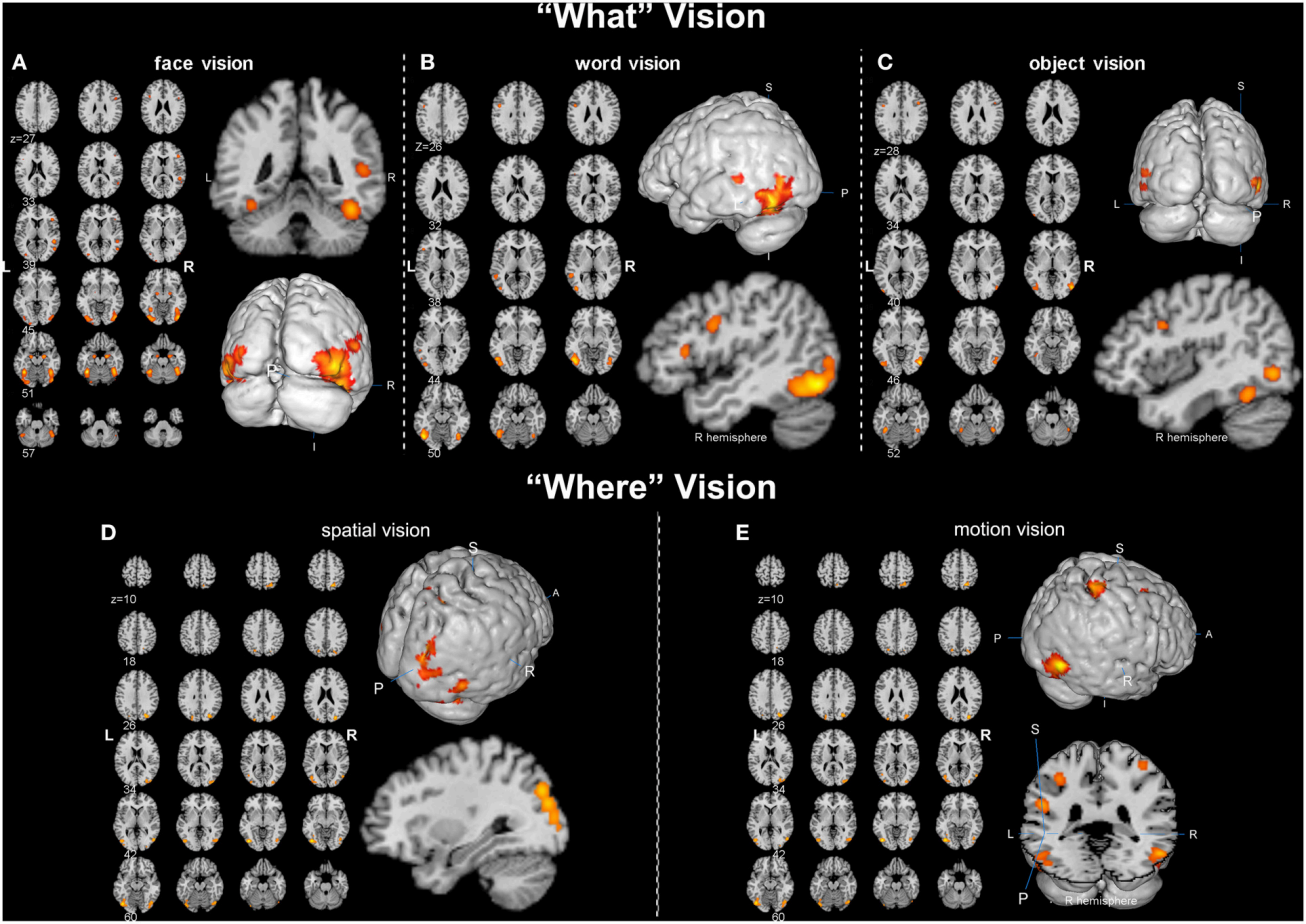
**Activation likelihood estimation (ALE) maps of “what” and “where” visions (*p* < 0.01, false discovery rate corrected). (A)** Left panel is the axial view of the ALE activation map for face vision. Lower right 3D image and upper coronal image show the significant clusters (in orange) in bilateral occipital lobe and right superior temporal sulcus. **(B)** Left panel is the axial view of the ALE activation map of word vision. Upper right 3D image and lower sagittal image show the significant clusters in the left inferior and middle temporal gyrus and the inferior frontal gyrus. **(C)** Left panel is the axial view of the ALE map for object vision. The magnified 3D and sagittal images display the significant clusters in the bilateral occipital lobe, right fusiform gyrus and inferior frontal gyrus. **(D)** Left panel is the axial view of the ALE activation map for spatial vision. Upper right 3D and lower sagittal images show the significant clusters in the right superior occipital gyrus and the right superior parietal lobule. **(E)** Left panel is the axial view of the ALE activation map for motion vision. The 3D images in the right panel display the significant clusters located in the bilateral tempo-occipital regions, right superior parietal gyrus, bilateral postcentral gyrus and left supramarginal gyrus.

**Figure 2:**
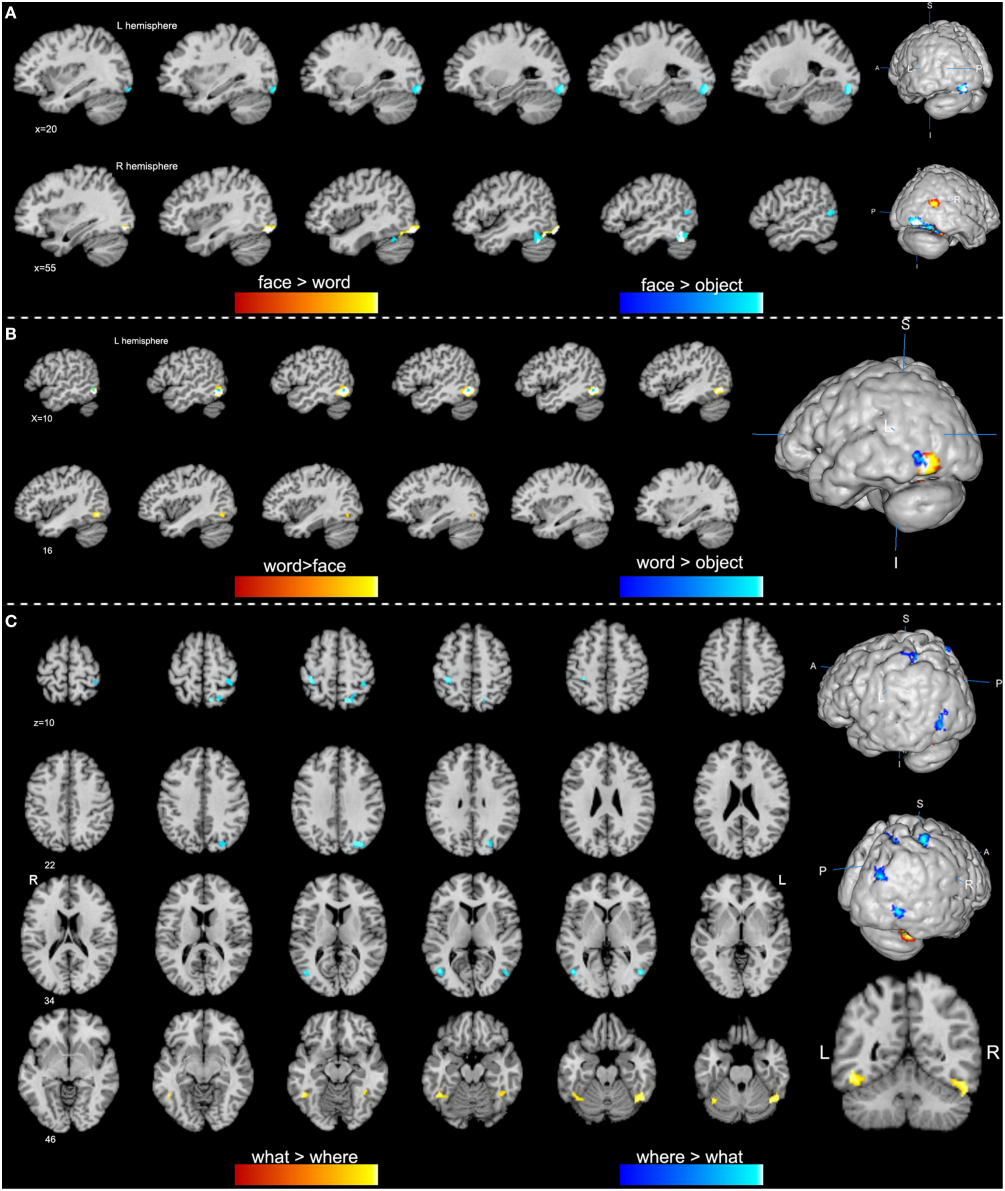
**Specific regions of high-level visions from contrast analyses (*p* < 0.01, false discovery rate corrected). (A)** Face-specific regions are obtained from the comparisons of face vs. word (orange) and face vs. object (blue). **(B)** Word-specific regions are obtained from contrast analyses of word vs. face (orange) and word vs. object (blue). **(C)** Clusters in orange indicate a higher activation of the “what” vision than “where” vision, while clusters in blue show a higher activation of the “where” vision.

**Figure 3:**
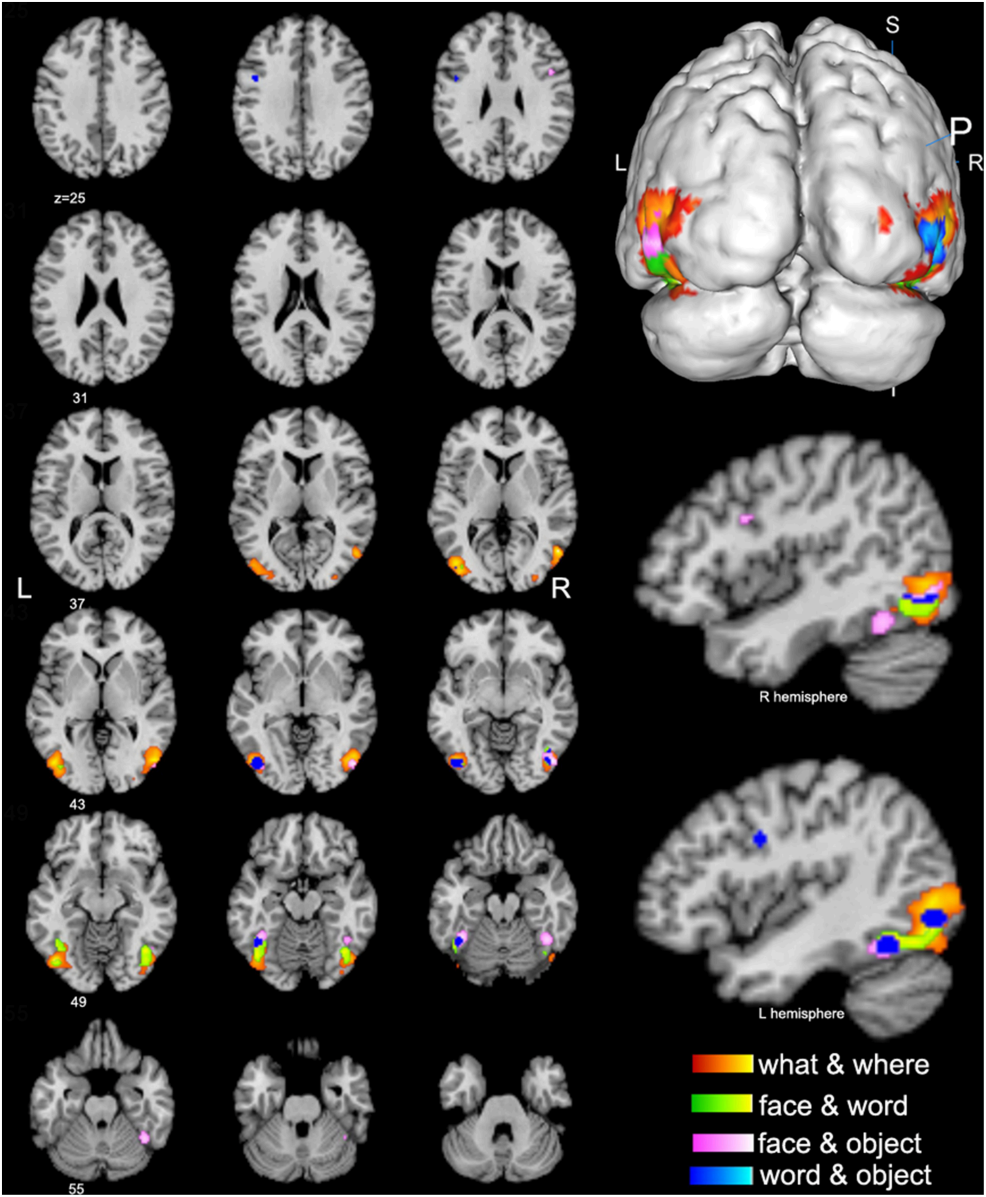
**Conjunctive regions of high-level visions (*p* < 0.01, false discovery rate corrected)**. The axial images in the left panel show the overview of the conjunctive regions for high-level visions of what & where (orange), face & word (green), face & object (pink), and word & object (blue). The magnified 3D image and the sagittal images in the right panel display that most of the conjunctive regions are convergently distributed in the bilateral lateral occipital complex (LOC).

Overall, convergent brain regions were identified from five ALE analyses of face, word, object, motion and spatial visual functions (Table 1, Figure 1) and were mainly distributed in the bilateral inferior and middle temporal gyrus (Brodmann area 19, BA 19) and the middle and inferior occipital gyrus (BA 18, 19). Moreover, the bilateral frontal lobes were found to be significantly activated, with regions for face, word, object and motion processing. Specific and conjunctive brain regions for different categories of visual functions acquired from contrast analyses are summarized in Table 2, Figures 2, 3.

**Table 1:** **Activation likelihood estimation (ALE) results of face, word, object, motion, and spatial vision**.

**Visual function**	**No. of experiments**	**No. of foci**	**No. of subjects**	**Cluster**	**Volume (mm^3^)**	**Peak activation coordinate (*x, y, z*)**	**Anatomic region (AAL)**	**Brodmann area**
Face vision	27	318	687	1	11040	46, −78, −12	R inferior occipital gyrus	19
						42, −48, −22	R fusiform gyrus	37
						42, −52, −20	R fusiform gyrus	37
				2	10256	−40, −54, −20	L fusiform gyrus	37
						−44, −80, −12	L inferior occipital gyrus	19
						−36, −86, −10	L middle occipital gyrus	19
				3	1944	20, −6, −20	R parahippocampal gyrus	28
				4	1416	−18, −8, −18	L hippocampus	35
				5	1232	50, −40, −8	R superior temporal gyrus	20
				6	792	46, 28, −10	R triangular part of the inferior frontal gyrus	47
				7	664	50,−40, 8	R middle temporal gyrus	21
				8	448	−22, −98, −4	L middle occipital gyrus	18
				9	280	46, 12, 26	R triangular part of inferior frontal gyrus	48
				10	216	56, −52, 14	R superior temporal gyrus	21
				11	208	−42, 18, 24	L triangular part of inferior frontal gyrus	48
Word/letter vision	16	119	211	1	7144	−46, −68, −12	L inferior temporal gyrus	19
						−48, −58, −16	L inferior temporal gyrus	37
						−44, −76, 2	L middle occipital gyrus	19
				2	1768	40, −62, −14	R inferior temporal gyrus	37
						42, −70, −14	R inferior temporal gyrus	19
				3	984	−56, −42, 2	L middle temporal gyrus	20
				4	560	−46, 4, 28	L opercular part of inferior frontal gyrus	44
				5	240	−48, 24, 10	L opercular part of inferior frontal gyrus	48
Object vision	18	112	251	1	2800	48, −72, −4	R middle temporal gyrus	19
						48, −62, −6	R middle temporal gyrus	37
				2	1320	−48, −74, −16	L middle occipital gyrus	19
				3	1080	−40, −50, −18	L fusiform gyrus	20
				4	1024	40, −50, −22	R fusiform gyrus	37
				5	328	42, 12, 30	R triangular part of inferior frontal gyrus	44
				6	296	−48, −82, 6	L middle occipital gyrus	19
				7	224	−46, 4, 30	L opercular part of inferior frontal gyrus	44
Spatial vision	30	247	376	1	5432	−44, −74, −8	L inferior occipital gyrus	19
						−46, −74, 2	L middle occipital gyrus	19
						−36, −86, 6	L middle occipital gyrus	18
						−40, −86, 4	L middle occipital gyrus	19
						−38, −58, −12	L fusiform gyrus	37
						−44, −66, 10	L middle temporal gyrus	37
				2	3864	28, −78, 32	R Superior occipital gyrus	19
						28, −82, 22	R middle occipital gyrus	19
						36, −88, 14	R middle occipital gyrus	19
						30, −88, 10	R middle occipital gyrus	18
				3	3088	52, −70, 2	R middle temporal gyrus	37
						44, −70, −16	R inferior temporal gyrus	19
						42, −72, −4	R middle temporal gyrus	19
						38, −84, −14	R inferior occipital gyrus	19
				4	1264	22, −62, 56	R superior parietal lobule	7
						10, −64, 62	R precuneus	
				5	696	−24, −80, 30	L superior occipital gyrus	17
						−26, −88, 26	L middle occipital gyrus	19
				6	368	−24, −70, 40	L superior parietal gyrus	18
Motion vision	14	205	221	1	3744	−50, −72, 4	L middle temporal gyrus	37
						−42, −82, 2	L middle occipital gyrus	19
						−46, −72, −12	L inferior occipital gyrus	19
				2	3416	50, −66, 2	R middle temporal gyrus	37
						46, −70, −12	R inferior temporal gyrus	19
				3	1392	−34, −36, 52	L postcentral gyrus	40
						−38, −38, 62	L postcentral gyrus	2
				4	1264	−50, −34, 26	L supramarginal gyrus	48
				5	816	34, −38, 64	R postcentral gyrus	3
				6	672	28, 0, 50	R middle frontal gyrus	6
				7	424	−26, −56, 60	L superior parietal	7

*Statistical level: p < 0.01; Minimum volume: 200 mm^3^; R-right, L-left*.

**Table 2:** **Contras analysis results between visual functions**.

	**Cluster**	**Volume (mm^3^)**	**Peak activation coordinate (*x, y, z*)**	**Anatomic region**	**Brodmann area**
**PART I CONTRAST**					
Face > Object	1	1936	41.41, −79.44, −15.66	R inferior occipital gyrus	19
			52, −58, −26	R inferior temporal gyrus	
	2	1224	−27.75, −87.23, −13.64	L inferior occipital gyrus	18
Face > Word	1	1224	49.18, −55.55, −26.29	R inferior temporal gyrus	37
			50, −61.33, −23.33	R inferior temporal gyrus	
	2	424	53.38, −60.53, 8.53	R middle temporal gyrus	37
Word > Face	1	2560	−50.67, −63.21, −10.61	L inferior temporal gyrus	37
Word > Object	1	208	−58, −60, −6	L middle temporal gyrus	37
			−50, −64, −10	L inferior temporal gyrus	37
			−58, −62, −10	L inferior temporal gyrus	37
Object > Face	1	376	46, −64, −3	R middle temporal gyrus	37
			50, −66, −4	R middle temporal gyrus	37
Object > Word	No significant higher activation				
“What” > “Where”	1	1808	46.68, −55.95, −25.49	R inferior temporal gyrus	37
	2	1168	−47.89, −51.78, −16.86	L inferior temporal gyrus	20
			−42, −54, −22	L fusiform gyrus	37
			−36, −56, −20	L fusiform gyrus	37
“Where” > “What”	1	1008	27.54, −76.75, 33.39	R superior occipital gyrus	19
	2	752	−47.54, −66.62, 5.38	L middle temporal gyrus	37
	3	736	−36.66, −34.26, 55.38	L precentral gyrus	3
	4	720	13.11, −63.15, 59.45	R superior parietal gyrus	7
			24, −59, 62	R superior parietal gyrus	7
			28, −60, 60	R superior parietal gyrus	7
	5	504	36.39, −38.56, 61.82	R postcentral gyrus	3
	6	464	47.05, −68.11, 3.16	R middle temporal gyrus	37
**PART II CONJUNCTION**					
Face & Object	1	1080	−40, −50, −18	L fusiform gyrus	37
	2	1204	40, −50, −22	R fusiform gyrus	37
	3	864	46, −74, −6	R middle temporal gyrus	19
			48, −62, −10	R inferior temporal gyrus	37
	4	296	−44, −76, −6	L middle occipital gyrus	19
Face & Word	1	2080	−46, −56, −16	L inferior temporal gyrus	37
			−44, −74, −10	L inferior occipital gyrus	19
			−42, −76, −8	L inferior occipital gyrus	19
			−42, −78, 0	L middle occipital gyrus	19
	2	1504	40, −62, −14	R inferior temporal gyrus	37
			42, −70, −14	R inferior temporal gyrus	19
Word & Object	1	840	−46, −76, −6	L middle occipital gyrus	19
	2	440	−44, −56, −18	L fusiform	37
“What” & “Where”	1	7584	−44, −72, −12	L inferior occipital gyrus	19
			−44, −76, 2	L middle occipital gyrus	19
			−42, −80, 4	L middle occipital gyrus	19
			−38, −60, −12	L inferior occipital gyrus	37
	2	5736	52, −64, 6	R middle temporal gyrus	37
			48, −70, −2	R middle temporal gyrus	37
			44, −70, −12	R inferior temporal gyrus	19
			44, −72, −4	R middle temporal gyrus	19
			36, −84, −16	R inferior occipital gyrus	19
	3	248	30, −88, 6	R middle occipital gyrus	18

*Statistical level: p < 0.01; Minimum volume: 200 mm^3^; R-right, L-left*.

#### Cortices corresponding to the five categories of visual functions

Our ALE analysis of face processing included 318 foci from 27 experiments. Eleven significant activation clusters extending across nine different BA in the bilateral temporal and occipital lobes, inferior frontal lobe and amygdala were found (Table 1, Figure 1A). Comparisons between face and word as well as between face and object processing showed higher activation for face processing in the bilateral occipital gyrus (BA 18), left inferior temporal gyrus (BA 19), and the right middle temporal/occipital gyrus (BA 37; Table 2, Figure 2A).

Sixteen experiments on word vision with 119 foci were included in our ALE meta-analysis, which revealed five significant clusters, including two in the bilateral inferior temporal gyrus (BA 19/37, both extending into the bilateral fusiform gyrus, Table 1, Figure 1B). The rest significant regions were found in the left middle temporal gyrus and the left opercular part of the inferior frontal gyrus, respectively. Contrast analyses of word vs. face and of word vs. object perception revealed that word-specific regions were mainly located in the left inferior temporal gyrus (BA 37) which extended to the middle temporal gyrus (BA 37; Table 2, Figure 2B).

In total, 112 foci from 18 experiments contributed to our ALE analysis of object perception. Seven significant clusters were identified (Table 1, Figure 1C), with five clusters located in the bilateral fusiform gyrus (BA 37), right middle temporal (BA 19/37) gyrus, and left middle occipital gyrus (BA 19). Two clusters were located in the bilateral inferior frontal gyrus (BA 44). Contrast analysis of object vs. face perception showed higher activation for object perception in the right middle temporal gyrus (BA 37), while there was no significant difference in activation for object perception when compared with word perception (Table 2).

Two hundred and forty-seven foci from 30 experiments contributed to our ALE analysis of spatial perception, and six clusters were found (Table 1, Figure 1D), which were mainly located in the temporal and occipital lobes (BA 18, 19, and 37). In addition, the bilateral superior parietal lobules were also found with significant clusters.

Our ALE analysis of motion perception included 205 foci from 14 experiments and found seven significant clusters (Table 1, Figure 1E). In addition to regions in the temporal and occipital lobes, significant regions were also found in the bilateral postcentral gyrus, left supramarginal gyrus, left superior parietal lobule (BA 7), and the right middle frontal gyrus (BA 6).

#### “what” vs. “where”

We performed contrast analyses broadly between the “what” and “where” visual streams to obtain specific brain regions. By comparison, the “what” stream showed overall higher activation in the bilateral inferior temporal gyrus and the left fusiform gyrus, while the “where” stream showed higher activation in the left precentral gyrus, right superior parietal lobule, middle occipital gyrus, middle temporal gyrus, and postcentral gyrus (Table 2, Figure 2C).

#### Conjunctively activated brain regions

The spatial distributions of brain regions with conjunctive activation acquired from the above contrast analyses were largely consistent and summarized here. The bilateral middle and inferior occipital gyrus, middle and inferior temporal gyrus, and fusiform gyrus were found to be the common conjunctive regions among most of the observed visual categories (Figure 3).

### Comparisons of GM volume

#### Demographics

There were no significant differences in gender, age, or education levels among the AD, EMCI, LMCI, and CN groups. Significant differences in MMSE scores were found among CN, EMCI, LMCI, and AD participants, and the AD patients performed the worst (Supplementary Table 2).

#### Volume differences in HLV cortices among groups

MANCOVA analysis revealed that there was significant difference in GM volume of the 36 ROIs among four groups (*p* = 9.07 × 10^−5^). By performing subsequent ANCOVA analyses, 25 ROIs were found with significantly different volume among groups (Table 3). *Post-hoc* analyses further revealed that the GM volume of 14 ROIs in the AD group was significantly smaller than in any other groups, and the volume of the rest 11 ROIs was significantly smaller than EMCI and CN groups (Figure 4). In LMCI subjects, only three clusters located in the left supramarginal gyrus, left middle, and inferior occipital gyrus were significantly atrophied compared with CN or EMCI (Figure 4). It's worth noting that all of the three clusters were involved in the motion and spatial visual processing. No clusters showed significant difference in GM volume between CN and EMCI subjects.

**Table 3:** **Mean volume differences of high-level visual cortices among groups**.

**ROIs' locations**	**Mean volume (±*SD*)**				***F*-value**	***p*-value**
	**CN**	**EMCI**	**LMCI**	**AD**		
**FACE VISION**						
R inferior occipital gyrus	5.44 ± 0.51	5.48 ± 0.54	5.30 ± 0.52	5.07 ± 0.7	3.61	0.003
L fusiform gyrus	5.32 ± 0.50	5.32 ± 0.50	5.10 ± 0.50	**4.77 ± 0.61**^^^	6.68	**2.015 × 10^−5^**
R parahippocampal gyrus	7.16 ± 0.72	7.17 ± 0.70	6.88 ± 1.33	6.05 ± 1.13^*^	8.55	**3.683 × 10^−6^**
R hippocampus	6.66 ± 0.70	6.59 ± 0.78	6.18 ± 1.19	5.33 ± 1.06^*^	13.11	**1.470 × 10^−8^**
R superior temporal gyrus	5.35 ± 0.77	5.46 ± 0.97	5.14 ± 0.92	4.55 ± 0.76^*^	9.23	**1.088 × 10^−4^**
R triangular part of inferior frontal gyrus	3.64 ± 0.41	3.82 ± 0.56	3.58 ± 0.49	3.44 ± 0.44	3.39	0.006
R middle temopral gyrus	5.28 ± 0.84	5.30 ± 0.88	4.87 ± 0.73	**4.61 ± 0.77**^^^	5.67	**6.883 × 10^−4^**
L middle occipital gyrus	3.36 ± 0.75	3.27 ± 0.85	3.32 ± 0.70	2.95 ± 0.66	2.44	0.103
R triangular part of inferior frontal gyrus	5.24 ± 0.82	5.48 ± 0.75	5.02 ± 0.83	**4.65 ± 0.74**^^^	7.50	**1.511 × 10^−4^**
R superior temporal gyrus	5.74 ± 0.74	5.96 ± 0.93	5.39 ± 0.58	**5.00 ± 0.74**^^^	8.88	**2.644 × 10^−6^**
L triangular part of inferior frontal gyrus	5.07 ± 0.82	5.54 ± 1.03	5.10 ± 0.90	4.53 ± 0.74^*^	6.36	**7.577 × 10^−5^**
**OBJECT VISION**						
R middle temporal gyrus	5.29 ± 0.64	5.21 ± 0.73	4.95 ± 0.62	**4.55 ± 0.65**^^^	6.61	**2.745 × 10^−5^**
L middle occipital gyrus	4.97 ± 0.61	4.85 ± 0.72	4.58 ± 0.59	**4.13 ± 0.92**^^^	7.83	**7.345 × 10^−6^**
L fusiform gyrus	6.90 ± 0.88	6.94 ± 0.75	6.65 ± 0.76	6.00 ± 0.86^*^	7.76	**7.802 × 10^−6^**
R fusiform gyrus	6.56 ± 0.82	6.70 ± 0.77	6.53 ± 0.66	6.21 ± 0.61	2.81	0.051
R triangular part of inferior frontalgyrus	5.53 ± 0.96	5.71 ± 0.87	5.22 ± 1.04	**4.72 ± 0.85**^^^	6.56	**8.222 × 10^−5^**
L middle occipital gyrus	3.39 ± 0.46	3.37 ± 0.53	3.23 ± 0.41	2.95 ± 0.73	4.86	0.001
L opercular part of inferior frontal gyrus	5.14 ± 0.82	5.20 ± 0.82	4.91 ± 0.95	4.45 ± 0.86	4.87	0.002
**WORD VISION**						
L inferior temporal gyrus	5.85 ± 0.60	5.72 ± 0.60	5.50 ± 0.53	4.89 ± 0.84^*^	11.39	**1.339 × 10^−8^**
R inferior temporal gyrus	5.79 ± 0.77	5.75 ± 0.71	5.48 ± 0.74	5.19 ± 0.67	4.37	0.002
L middle temporal gyrus	6.00 ± 0.66	6.10 ± 1.00	5.60 ± 1.00	4.72 ± 1.02^*^	13.26	**4.792 × 10^−9^**
L opercular part of inferior frontal gyrus	4.79 ± 0.63	4.89 ± 0.64	4.59 ± 0.81	**4.22 ± 0.69**^^^	6.39	**3.873 × 10^−4^**
L opercular part of inferior frontal gyrus	4.53 ± 0.66	4.71 ± 0.74	4.63 ± 0.74	4.16 ± 0.83	2.95	0.016
**MOTION VISION**						
L middle occipital gyrus	5.12 ± 0.61	5.05 ± 0.68	**4.66 ± 0.52**^#^	**4.26 ± 0.88**^^^	9.24	**3.025 × 10^−7^**
R middle temporal gyrus	5.45 ± 0.78	5.33 ± 0.79	5.05 ± 0.66	**4.73 ± 0.64**^^^	6.01	**2.746 × 10^−4^**
L postcentral gyrus	4.09 ± 0.66	4.27 ± 0.67	4.17 ± 0.58	3.59 ± 0.68^*^	75.95	**1.355 × 10^−4^**
L supramarginal gyrus	4.52 ± 0.49	4.60 ± 0.73	**4.08 ± 0.58**^^^	**3.98 ± 0.58**^^^	7.45	**6.385 × 10^−6^**
R postcentral gyrus	3.27 ± 0.57	3.27 ± 0.50	3.27 ± 0.56	2.92 ± 0.54	2.74	0.021
R middle frontal gyrus	5.10 ± 0.78	5.31 ± 1.13	4.98 ± 0.58	4.65 ± 0.80	3.41	0.009
R superior parietal gyrus	4.47 ± 0.88	4.70 ± 0.91	4.74 ± 0.65	3.86 ± 0.88^*^	5.71	**8.849 × 10^−5^**
**SPATIAL VISION**						
L inferior occipital gyrus	5.16 ± 0.49	5.06 ± 0.53	**4.80 ± 0.49**^#^	4.39 ± 0.79^*^	9.56	**1.740 × 10^−7^**
R superior occipital gyrus	4.42 ± 0.56	4.47 ± 0.61	4.31 ± 0.52	3.82 ± 0.61^*^	6.93	**2.054 × 10^−5^**
R middle temporal gyrus	5.11 ± 0.63	5.07 ± 0.59	4.88 ± 0.61	4.60 ± 0.60	4.37	0.001
R superior parietal lobule	4.32 ± 0.64	4.40 ± 0.83	4.37 ± 0.56	3.56 ± 0.71^*^	8.38	**1.518 × 10^−6^**
L superior occipital gyrus	3.81 ± 0.60	3.88 ± 0.53	3.90 ± 0.58	3.28 ± 0.76^*^	5.77	**9.225 × 10^−5^**
L superior parietal gyrus	4.84 ± 0.79	5.03 ± 0.81	4.78 ± 0.63	4.09 ± 1.02^*^	6.61	**2.408 × 10^−5^**

*Statistical level: p < 0.0014 (Bonferroni correction)*.

* *Significantly different from other groups in Post-hoc analysis (p < 0.05, Bonferroni correction)*.

^ *Significantly different from the CN and EMCI group in Post-hoc analysis (p < 0.05, Bonferroni correction)*.

# *Significantly different from the CN group in Post-hoc analysis (p < 0.05, Bonferroni correction)*.

**Figure 4:**
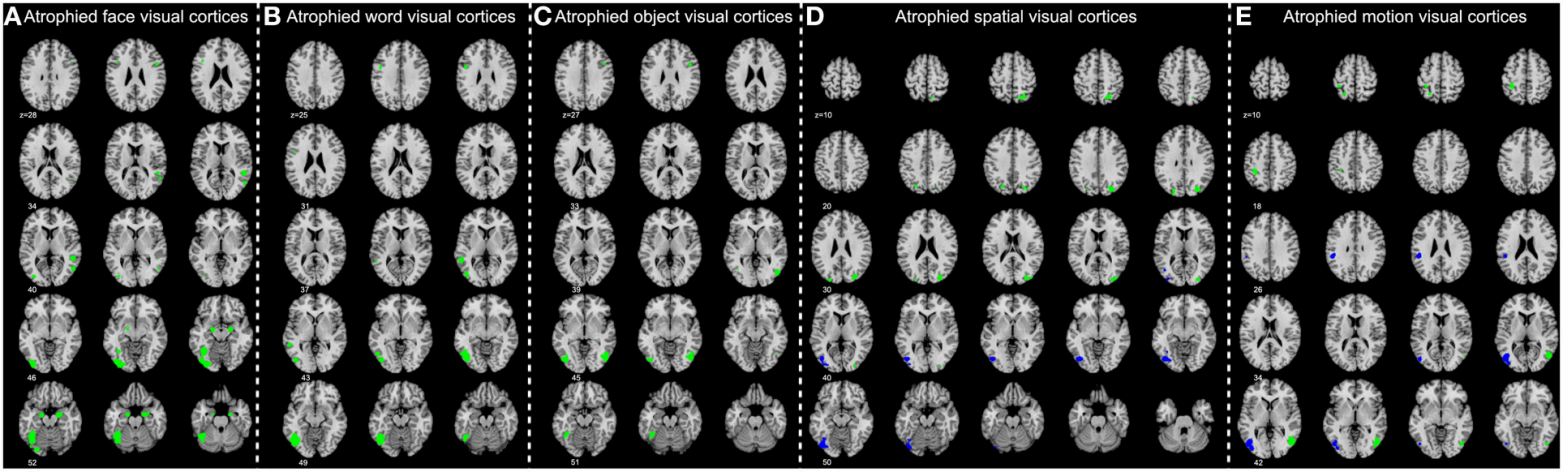
**Significantly atrophied high-level visual cortices in patients with Alzheimer's disease (AD) and mild cognitive impairment (MCI)**. The figure shows high-level visual regions of interest (ROIs) with significantly reduced mean gray matter volume in AD and late MCI (LMCI) groups. These ROIs are respectively corresponding to the face **(A)**, word **(B)**, object **(C)**, motion **(D)**, and spatial **(E)** visual processing. Regions in green color are found significantly atrophied only in AD group, while regions in blue are found significantly atrophied in both AD and LMCI groups.

#### Correlation analyses results

The correlation results between GM volume of the 25 ROIs with significant atrophy and the cognitive test scores and whole-brain mean GM volume are summarized in Table 4. Overall, both the MMSE score and whole-brain mean GM volume were found with a significantly positive correlation to the regional GM volume in most of the 25 ROIs. In addition, the performances of category fluency-animal and trail making test A & B were found to be positively correlated with the GM volume in most ROIs of both “what” and “where” visual cortices. For the logical memory test I & II and Boston naming test, significant correlation between GM volume and test performances was found to be more widespread in “what” visual cortices than in “where” visual cortices. Whereas, in clock drawing test, significant correlation between GM volume and test score was only found in a small portion of ROIs.

**Table 4:** **Correlation of gray matter (GM) volume of the atrophied visual cortices in patient groups to cognitive tests and whole-brain mean GM volume**.

**Locations of ROIs (AAL)**	**Clock drawing test**		**Logical memory test I**		**Logical memory test II**		**Category fluency-animal**		**Trail making test A**		**Trail making test B**		**Boston naming test**		**MMSE**		**GM volume**	
	***r***	***p***	***r***	***p***	***r***	***p***	***r***	***p***	***r***	***p***	***r***	***p***	***r***	***p***	***r***	***p***	***r***	***p***
**FACE VISION**																		
L fusiform gyrus	0.21	0.007	0.34	**7.69 × 10^−4^**	0.36	**4.38 × 10^−6^**	0.31	**6.58 × 10^−5^**	–0.34	**1.37 × 10^−5^**	–0.37	**2.91 × 10^−6^**	0.37	**1.70 × 10^−6^**	0.29	**2.41 × 10^−4^**	0.41	**9.06 × 10^−8^**
R parahippocampal gyrus	0.15	0.062	0.29	**2.51 × 10^−4^**	0.34	**1.49 × 10^−5^**	0.22	0.005	–0.30	**1.29 × 10^−4^**	–0.33	**3.59 × 10^−5^**	0.30	**1.42 × 10^−4^**	0.31	**7.09 × 10^−5^**	0.26	**0.001**
R hippocampus	0.19	0.015	0.39	**4.63 × 10^−7^**	0.43	**2.09 × 10^−8^**	0.26	**0.001**	–0.33	**1.55 × 10^−5^**	–0.39	**6.67 × 10^−7^**	0.34	**8.31 × 10^−6^**	0.39	**2.48 × 10^−7^**	0.25	**0.0015**
R superior temporal gyrus	0.29	**2.13 × 10^−4^**	0.33	**1.78 × 10^−5^**	0.33	**2.38 × 10^−5^**	0.29	**2.10 × 10^−4^**	–0.31	**7.94 × 10^−5^**	–0.41	**1.26 × 10^−7^**	0.34	**8.50 × 10^−6^**	0.39	**2.92 × 10^−7^**	0.12	0.12
R middle temporal gyrus	0.24	**0.0018**	0.21	0.009	0.20	0.010	0.28	**2.73 × 10^−4^**	–0.33	**1.99 × 10^−5^**	–0.32	**7.86 × 10^−5^**	0.26	**0.001**	0.24	0.002	0.23	0.003
R triangular part of inferior frontal gyrus	0.16	0.045	0.24	0.003	0.25	**0.001**	0.23	0.003	–0.28	**3.06 × 10^−4^**	–0.27	**0.001**	0.15	0.065	0.30	**1.36 × 10^−4^**	0.17	0.03
R superior temporal gyrus	0.14	0.070	0.22	0.005	0.29	**2.61 × 10^−4^**	0.28	**2.80 × 10^−4^**	–0.25	**0.0016**	–0.25	**0.0017**	0.29	**3.60 × 10^−4^**	0.33	**2.15 × 10^−5^**	0.22	0.006
L triangular part of inferior frontal gyrus	0.31	**4.88 × 10^−5^**	0.22	0.005	0.20	0.012	0.22	0.005	–0.18	0.027	–0.26	**0.0015**	0.18	0.021	0.28	**2.94 × 10^−4^**	0.27	**6.02 × 10^−4^**
**OBJECT VISION**																		
R middle temporal gyrus	0.24	**0.0019**	0.27	**0.001**	0.28	**3.32 × 10^−4^**	0.33	**1.61 × 10^−5^**	–0.35	**5.85 × 10^−5^**	–0.46	**2.73 × 10^−9^**	0.29	**1.71 × 10^−4^**	0.29	**1.57 × 10^−4^**	0.32	**4.01 × 10^−5^**
L middle occipital gyrsu	0.25	**0.001**	0.36	**2.58 × 10^−6^**	0.35	**5.53 × 10^−6^**	0.29	**1.59 × 10^−4^**	–0.33	**2.36 × 10^−5^**	–0.38	**1.28 × 10^−6^**	0.30	**1.13 × 10^−4^**	0.29	**2.27 × 10^−4^**	0.26	**8.84 × 10^−4^**
L fusiform gyrsu	0.20	0.009	0.35	**8.28 × 10^−6^**	0.35	**6.29 × 10^−6^**	0.34	**8.45 × 10^−6^**	–0.30	**1.24 × 10^−4^**	–0.39	**8.00 × 10^−7^**	0.37	**1.39 × 10^−6^**	0.30	**1.45 × 10^−4^**	0.28	**2.95 × 10^−4^**
R triangular part of inferior frontal gyrus	0.19	0.014	0.24	0.002	0.27	0.001	0.27	**0.001**	–0.22	0.005	–0.29	**2.58 × 10^−5^**	0.17	0.030	0.30	**1.27 × 10^−4^**	0.22	0.006
**WORD VISION**																		
L inferior temporal gyrus	0.28	**2.59 × 10^−4^**	0.43	**1.26 × 10^−8^**	0.43	**1.49 × 10^−8^**	0.39	**3.03 × 10^−7^**	–0.42	**2.29 × 10^−8^**	–0.48	**3.35 × 10^−10^**	0.41	**5.40 × 10^−8^**	0.35	**7.28 × 10^−6^**	0.37	**1.47 × 10^−6^**
L middle temporal gyrus	0.21	0.007	0.37	**2.20 × 10^−6^**	0.39	**4.85 × 10^−7^**	0.40	**1.04 × 10^−7^**	–0.29	**2.07 × 10^−5−−4^**	–0.35	**7.96 × 10^−6^**	0.26	**0.001**	0.39	**4.57 × 10^−7^**	0.28	**2.87 × 10^−4^**
L opercular part of inferior frontal gyrus	0.17	0.023	0.29	**1.82 × 10^−4^**	0.07	0.373	0.29	**1.50 × 10^−4^**	–0.24	0.002	–0.20	0.015	0.16	0.048	0.31	**7.45 × 10^−5^**	0.18	0.02
**MOTION VISION**																		
L middle occipital gyrus	0.33	**1.85 × 10^−5^**	0.32	**3.02 × 10^−5^**	0.32	**5.20 × 10^−4^**	0.34	**1.07 × 10^−5^**	–0.38	**8.06 × 10^−7^**	–0.43	**3.24 × 10^−8^**	0.34	**1.26 × 10^−5^**	0.32	**4.55 × 10^−6^**	0.37	**1.16 × 10^−6^**
R middle temporal gyrsu	0.28	**3.22 × 10^−4^**	0.23	0.004	0.24	0.003	0.31	**6.35 × 10^−5^**	0.35	**4.34 × 10^−6^**	–0.43	**4.87 × 10^−8^**	0.29	**2.46 × 10^−4^**	0.28	**4.05 × 10^−4^**	0.25	**0.0016**
L postcentral gyrus	0.15	0.067	0.15	0.061	0.18	0.024	0.26	**0.001**	–0.34	**1.41 × 10^−5^**	–0.34	**2.11 × 10^−5^**	0.19	0.014	0.25	**0.001**	0.24	0.002
L supramarginal gyrus	0.24	0.002	0.41	**9.83 × 10^−8^**	0.33	**2.11 × 10^−4^**	0.37	**1.08 × 10^−6^**	–0.34	**1.05 × 10^−5^**	–0.36	**5.19 × 10^−6^**	0.27	**0.001**	0.28	**2.95 × 10^−4^**	0.28	**3.45 × 10^−4^**
R superior parietal gyrus	0.18	0.024	0.08	0.318	0.15	0.056	0.18	0.021	–0.27	**0.001**	–0.25	**0.0019**	0.15	0.062	0.27	**5.56 × 10^−4^**	0.27	**4.80 × 10^−4^**
**SPATIAL VISION**																		
L inferior occipital gyrus	0.29	**2.19 × 10^−4^**	0.39	**3.18 × 10^−7^**	0.39	**3.90 × 10^−7^**	0.33	**1.83 × 10^−5^**	–0.41	**9.29 × 10^−8^**	–0.40	**2.96 × 10^−7^**	0.37	**1.73 × 10^−6^**	0.31	**4.66 × 10^−5^**	0.40	**1.68 × 10^−7^**
R superior occipital gyrus	0.26	**0.001**	0.23	0.003	0.26	**0.001**	0.28	**2.86 × 10^−4^**	–0.32	**4.14 × 10^−5^**	–0.32	**6.00 × 10^−5^**	0.17	0.034	0.34	**8.37 × 10^−6^**	0.35	**6.19 × 10^−6^**
R superior parietal lobule	0.22	0.005	0.16	0.046	0.21	0.007	0.26	**0.001**	–0.30	**1.56 × 10^−4^**	–0.28	**4.08 × 10^−4^**	0.18	0.020	0.31	**7.21 × 10^−5^**	0.34	**1.06 × 10^−5^**
L superior occipital gyrus	0.20	0.013	0.16	0.046	0.19	0.020	0.25	**0.0015**	–0.30	**1.15 × 10^−4^**	–0.31	**1.12 × 10^−4^**	0.09	0.265	0.25	**0.001**	0.28	**3.03 × 10^−4^**
L superior parietal lobule	0.23	0.003	0.20	0.013	0.22	0.007	0.20	0.010	–0.26	**0.001**	–0.20	0.016	0.07	0.351	0.25	**0.001**	0.26	**7.88 × 10^−4^**

L-left; R-right;

Statistical level: p < 0.002;

*MMSE: mini-mental state examination*.

## Discussion

In this study, we performed a series of ALE analyses to map the “what” and “where” visual cortices, and we systematically differentiated them with regard to five categories visual perceptions: object, face, word, motion, and spatial. Subsequently, brain areas with specific and conjunctive activation for processing these visual functions were identified by contrast analyses. Finally, based upon the ALE-defined spatial locations, the atrophy patterns of these HLV cortices in MCI and AD patients were evaluated. The results showed that the distribution of atrophied brain regions was widespread in AD patients and localized in MCI subjects, and these observed findings were related to the deterioration of overall cognitive status. To our knowledge, this is the first comprehensive coordinate-based meta-analysis of “what” and “where” visual perceptions, and also the first morphometric analysis focusing on the HLV cortices in AD and MCI patients.

### The HLV networks of the brain for “what” and “where” visions

Our whole-brain ALE analyses suggested a series of brain regions that were involved in the HLV functions of “what” and “where” visions. On one hand, convergent distributions of these clusters were found in the occipital and temporal gyrus, which were consistent with the locations of the traditionally considered brain components for HLV processing in previous findings (Grill-Spector and Malach, 2004; Wandell et al., 2007). On the other hand, the bilateral inferior frontal gyrus and superior parietal lobule were found to be significant for most of the “what” and “where” visual processes, respectively, perhaps underlying their involvement in HLV processing. In particular, the inferior frontal gyrus was found to be involved in emotional responses, riskier choices and language comprehension (Sakai, 2005; Vigneau et al., 2006; Sarubbo et al., 2013). The superior parietal lobule plays a pivotal role in many sensory and cognitive processes, including spatial perception (Faillenot et al., 1997; Weiss et al., 2003), integration for motor acts (Culham et al., 2006), motor learning (Weiss et al., 2003) and visuospatial attention (Corbetta et al., 1995), etc. In addition, in Kravitz's review (Kravitz et al., 2013), they also described the superior parietal lobule contains 3D representation of objects for visuospatial processing and closely interconnected with occipital and prefrontal regions to form a framework of visuospatial processing. Hence, we assume that the inferior frontal gyrus and the superior parietal lobule may collaborate with regions in the occipital and temporal lobes to exercise particularly HLV processing; thus, these two regions may also be included as components constituting a HLV network. These findings are also supported by those of previous studies (de Haan and Cowey, 2011; Kravitz et al., 2013). However, it is worth noting that the current finding in the inferior frontal gyrus and superior parietal lobule may also possibly due to a general response to the tasks (e.g., memory and attention, etc.). Thus, further studies on whether the two regions are activated at the visual perceptual stage or at the subsequent information integration or action guidance stage are encouraged to shed light on this question.

### Specific brain areas for high-level vision

Contrast analyses were used to investigate specific brain regions for particular visual functions. When compared with face and object visions, the brain regions specific for word processing were found in the left inferior temporal gyrus. This result is in accordance with previous evidence that specific activation by word or letter stimuli locates in the left inferior occipito-temporal cortex of the human brain (Cohen et al., 2002; Glezer et al., 2009). It's worth mentioning that to maintain the homogeneity of the meta-analysis, we only included studies of alphabetic word perception. Based on the fact that only a few studies of logographic words are available, it would be interesting for future comparative meta-analyses to investigate specific brain areas for perceiving words in different languages and writing systems.

Three of the “face specific” clusters revealed in our study were located in the inferior temporal (BA 19/37) and occipital gyrus (BA 18/19). These anatomical locations correspond well to the previously defined occipital face area (OFA; Yovel and Kanwisher, 2004), which are thought to be specialized in facial information processing. In addition, the posterior portion of right middle temporal gyrus was found as another “face specific” region when compared with word vision. There is also previous evidence that supported this area as a face processing activated region (Hein and Knight, 2008). However, the “fusiform face area,” which is recognized as one of the classical face processing regions, was not found in our contrast result. Alternatively, this region was shown as a conjunctive region for both face and object processing. In Haxby's and Hanson's studies (Haxby et al., 2001; Hanson et al., 2004), they found that the representations of faces and objects in the ventral temporal cortex are highly overlapping, thus, the specificity of cortical response to certain visual category may be difficult to be clarified in this area. Therefore, taken together with our results, we may speculate that the fusiform gyrus may contribute to both face and non-living object recognition. Nevertheless, future fMRI studies with higher spatial and temporal resolution are still required to provide more findings on the specificity of fusiform gyrus in recognition perception.

The contrasts between the two major aspects of HLV functions, i.e., the “what” and “where” streams, revealed that “where” visual perception was related to greater activation in a series of brain regions, including the superior occipital gyrus, superior parietal lobule, posterior portion of middle temporal gyrus, precentral and postcentral gyrus. The results were consistent with the distribution of brain regions that formed the dorsal visual pathway, as described in Kravitz's review (Kravitz et al., 2011). Besides, these brain regions were considered closely interconnected and formed a framework to support the spatial and motion perception. In contrast, “what” visual perception showed a greater involvement of the bilateral inferior temporal gyrus. In accordance with previous findings, this result highlights the role of inferior temporal gyrus in “what” vision processing (Kravitz et al., 2013).

### Conjunctive regions for high-level visual functions

Conjunctive brain regions acquired from contrast analyses among different visual categories were convergently distributed in the right inferior and middle temporal gyrus (BA 19/37) and bilateral middle and inferior occipital gyrus (BA 18, 19, 37), which essentially correspond to the location of the lateral occipital complex (LOC). The LOC has been considered to be the prime locus of object identity representation (Ishai et al., 2000; Cichy et al., 2011), and it is traditionally considered as a part of the “what” visual processing stream. One possible reason for its conjunctive activation in terms of “where” visual processing may be that most spatial and motion visual tasks use certain objects or forms as visual targets, and object identification may be constantly included as a precursor for “where” processing. Therefore, our current findings in the LOC may indicate the existence of interrelationships between “what” and “where” information processing (i.e., the functional crosstalk between the ventral and dorsal stream) and contribute to our understanding of this type of crosstalk.

### Atrophied HLV cortices in AD and MCI

Our morphometric analyses between AD and CN revealed that the atrophy of HLV cortices in AD was widespread. Most of the cortices responsible for both the “what” (i.e., face, object and word visions) and “where” (i.e., motion and spatial visions) visual functions were involved. Previous neuropsychological and fMRI findings have also provided evidence that visual impairments in AD patients arise from both the ventral and dorsal streams (Kim and Park, 2010; Kirby et al., 2010). In addition, compared with controls, more amyloid deposits (one of the major reason for neuronal cell death) were found distributed in parietal, temporal, occipital and frontal regions, and these were related to the impairment of HLV functions (Edison et al., 2007; Rodrigue et al., 2009) in AD patients. These pathological findings further support our results and taken together provide a neurobiological basis for disrupted HLV functions in AD.

In addition, our results may support that the atrophy of HLV cortices develops at a late stage of the disease (i.e., AD/LMCI), because no significant differences were found between EMCI and CN. This might indicate that the atrophy of HLV cortices could be a late-stage outcome of Alzheimer's disease progression. In addition, the distribution of atrophied visual cortices in LMCI patients was comparatively localized (with only three significant regions), while much more widespread in AD patients (with 25 significant regions). Besides, the correlation results also suggested that the observed atrophy in HLV cortices was correlated to the whole GM atrophy and the deterioration of overall cognitive status in MCIs and ADs. Therefore, these finding may indicate that the atrophy of HLV cortices could be in accordance with the AD-related pathological progression. Thus, the extent of the visual cortical atrophy may be of some indicative value for the severity and disease progression of AD.

Meanwhile, it's worth noting that the correlation between MMSE and GM atrophy is found to be weak in most ROIs (with the *r*-values around 0.2–0.4). This may indicate that the HLV cortices changes are not entirely dependent on the whole cortical atrophy or disease severity of AD and MCI. Alternatively, it may also reflect and be related to some intrinsic or independent alteration pattern of the HLV network *per se*. In line with this, our results also revealed differential correlations between GM volume of HLV cortices and the cognitive tests of different domains. For example, the observed brain atrophy was found to be related to clock drawing test score in a few HLV regions, while was widely correlated to the performances of category fluency-animal and trail making test A & B (with the *r*-values around 0.3–0.4). This perhaps underlies a comparatively close relationship between HLV cortical atrophy and the impaired sematic memory and executive function in ADs and MCIs. In addition, for the logical memory test I & II and Boston naming test, it's worth noting that association between GM atrophy and the test performances was found to be more widespread in “what” visual cortices than in “where visual” cortices. On one hand, it is anticipated that Boston naming test score is closely related to the impairment of “what” visual cortices since it involves object recognition function which is primarily processed by ventral stream. On the other hand, findings in logical memory test may also provide evidence to support that atrophied “what” visual cortices may have an close interplay with the memory dysfunction under AD-related neuropathology. However, the specific causal relationship is to be investigated.

Furthermore, another interesting finding was that all of the three significant atrophied regions in LMCI group were included in the “where” visual cortices (i.e., regions related to motion and spatial visual functions), while no significant atrophy was found in the “what” visual regions. It's worth noting that a similar selective impairment pattern of “where” visual cortices in AD has also been reported by previous studies (Mandal et al., 2012). Taken together, we might speculate that “where” visual cortices are perhaps more vulnerable to the AD-related neuropathological changes than “what” visual cortices.

### Limitations

Several limitations in the study should be noted. First, the number of included studies for ALE analysis is relatively low. This was mainly due to our criterion that only studies using low-level contrasts were included. It should be noted that most of the previous studies on HLV functions were performed with various types of high-level contrasts, which may be related to heterogeneous brain stimuli. Therefore, although the current inclusion strategy might largely limit the sample size, it will help maintain the homogeneity of meta-analysis. Meanwhile, it should be noted that the number of studies among different visual functions for some contrast analyses was not equivalent. Thus, the statistical power of these contrasts may have been affected. Second, the literature coverage was restricted to studies investigating HLV functions of the “what” and “where” streams, which led to only five categories of visual perception with comparatively large sample sizes. Further meta-analyses focusing on other aspects of the HLV functions will be encouraged to accomplish a more comprehensive probabilistic HLV atlas. Third, to enable a comparison between current VBM results and functional findings in the future study, we only chose the subjects both with 3D T1WI and resting-state fMRI data in the ADNI dataset to increase the comparability. Therefore, the subject number of 161, though acceptable, may be relatively small compared with the whole ADNI dataset. However, it should be noted that the MRI data in ADNI dataset were obtained from different scanners (Philips, GE, or Seimens) with different field strengths (3.0T or 1.5T), while our selected data group would only include those obtained with 3T Philips scanners. Therefore, our current data selection strategy also helped to control the influence of scanning factors.

## Conclusions

In summary, this study combined ALE and VBM analyses to map the HLV cortices and their atrophy in AD progression. The ALE results showed brain regions for processing the visual perceptions of different categories included in the “what” and “where” visions, as well as their conjunctions and specifications. Based on the ALE maps, comparison results of GM volume demonstrated atrophy of HLV cortices in AD and LMCI individuals. In addition, the observed atrophy was correlated to the deterioration of overall cognitive status and to the cognitive domains of memory, execution, and object recognition functions. These results possibly indicate that the visual cortical atrophy may be of some indicative value for the disease severity and progression of AD. Therefore, these findings may enrich our understanding of the organizational patterns of HLV networks in the human brain, and it may also extend our knowledge on the evolvement of visual perceptual dysfunction in AD progression. Moreover, we hope our results could be applied in further studies (e.g., studies investigating functional connectivity based on our coordinates of HLV cortices) of HLV cortices and related diseases.

## Author contributions

YD: design of the work, data analysis, manuscript writing, account for all aspects of the work; LS: final article approval; YL: accountable for all aspect of the work; PL: accountable for all aspect of the work; KL: accountable for all aspect of the work; WC: accountable for all aspect of the work; DW: final article approval.

### Conflict of interest statement

The authors declare that the research was conducted in the absence of any commercial or financial relationships that could be construed as a potential conflict of interest.

## References

[B1] Adlington R. L. Laws K. R. Gale T. M. (2009). Visual processing in Alzheimer's disease: surface detail and colour fail to aid object identification. Neuropsychologia 47, 2574–2583. 10.1016/j.neuropsychologia.2009.05.004 19450614

[B2] Ashburner J. (2007). A fast diffeomorphic image registration algorithm. Neuroimage 38, 95–113. 10.1016/j.neuroimage.2007.07.007 17761438

[B3] Backus B. T. Fleet D. J. Parker A. J. Heeger D. J. (2001). Human cortical activity correlates with stereoscopic depth perception. J. Neurophysiol. 86, 2054–2068. 11600661 10.1152/jn.2001.86.4.2054

[B4] Cichy R. M. Chen Y. Haynes J. D. (2011). Encoding the identity and location of objects in human LOC. Neuroimage 54, 2297–2307. 10.1016/j.neuroimage.2010.09.044 20869451

[B5] Cohen L. Lehericy S. Chochon F. Lemer C. Rivaud S. Dehaene S. (2002). Language-specific tuning of visual cortex? Functional properties of the Visual Word Form Area. Brain 125, 1054–1069. 10.1093/brain/awf094 11960895

[B6] Corbetta M. Shulman G. L. Miezin F. M. Petersen S. E. (1995). Superior parietal cortex activation during spatial attention shifts and visual feature conjunction. Science 270, 802–805. 10.1126/science.270.5237.802 7481770

[B7] Culham J. (2015). Visuomotor integration, in Brain Mapping An Encyclopedic Reference, ed Toga A. W. (Vienna: Elsevier), 469–474.

[B8] Culham J. C. Cavina-Pratesi C. Singhal A. (2006). The role of parietal cortex in visuomotor control: what have we learned from neuroimaging? Neuropsychologia 44, 2668–2684. 10.1016/j.neuropsychologia.2005.11.003 16337974

[B9] de Haan E. H. Cowey A. (2011). On the usefulness of ‘what’ and ‘where’ pathways in vision. Trends Cogn. Sci. 15, 460–466. 10.1016/j.tics.2011.08.005 21906989

[B10] Delbeuck X. Van der Linden M. Collette F. (2003). Alzheimer's disease as a disconnection syndrome? Neuropsychol. Rev. 13, 79–92. 10.1023/A:1023832305702 12887040

[B11] Downing P. E. Chan A. W. Peelen M. V. Dodds C. M. Kanwisher N. (2006). Domain specificity in visual cortex. Cereb. Cortex 16, 1453–1461. 10.1093/cercor/bhj086 16339084

[B12] Edison P. Archer H. A. Hinz R. Hammers A. Pavese N. Tai Y. F. (2007). Amyloid, hypometabolism, and cognition in Alzheimer disease: an [11C]PIB and [18F]FDG PET study. Neurology 68, 501–508. 10.1212/01.wnl.0000244749.20056.d4 17065593

[B13] Eickhoff S. B. Bzdok D. Laird A. R. Kurth F. Fox P. T. (2012). Activation likelihood estimation meta-analysis revisited. Neuroimage 59, 2349–2361. 10.1016/j.neuroimage.2011.09.017 21963913 PMC3254820

[B14] Eickhoff S. B. Laird A. R. Grefkes C. Wang L. E. Zilles K. Fox P. T. (2009). Coordinate-based activation likelihood estimation meta-analysis of neuroimaging data: a random-effects approach based on empirical estimates of spatial uncertainty. Hum. Brain Mapp. 30, 2907–2926. 10.1002/hbm.20718 19172646 PMC2872071

[B15] Eslinger P. J. Damasio A. R. Benton A. L. Van Allen M. (1985). Neuropsychologic detection of abnormal mental decline in older persons. JAMA 253, 670–674. 10.1001/jama.1985.03350290076029 3968802

[B16] Faillenot I. Toni I. Decety J. Gregoire M. C. Jeannerod M. (1997). Visual pathways for object-oriented action and object recognition: functional anatomy with PET. Cereb. Cortex 7, 77–85. 10.1093/cercor/7.1.77 9023435

[B17] Glezer L. S. Jiang X. Riesenhuber M. (2009). Evidence for highly selective neuronal tuning to whole words in the “visual word form area”. Neuron 62, 199–204. 10.1016/j.neuron.2009.03.017 19409265 PMC2706007

[B18] Grill-Spector K. Knouf N. Kanwisher N. (2004). The fusiform face area subserves face perception, not generic within-category identification. Nat. Neurosci. 7, 555–562. 10.1038/nn1224 15077112

[B19] Grill-Spector K. Malach R. (2004). The human visual cortex. Annu. Rev. Neurosci. 27, 649–677. 10.1146/annurev.neuro.27.070203.144220 15217346

[B20] Hanson S. J. Matsuka T. Haxby J. V. (2004). Combinatorial codes in ventral temporal lobe for object recognition: haxby (2001) revisited: is there a “face” area? Neuroimage 23, 156–166. 10.1016/j.neuroimage.2004.05.020 15325362

[B21] Haxby J. V. Gobbini M. I. Furey M. L. Ishai A. Schouten J. L. Pietrini P. (2001). Distributed and overlapping representations of faces and objects in ventral temporal cortex. Science 293, 2425–2430. 10.1126/science.1063736 11577229

[B22] Haxby J. V. Grady C. L. Horwitz B. Ungerleider L. G. Mishkin M. Carson R. E.. (1991). Dissociation of object and spatial visual processing pathways in human extrastriate cortex. Proc. Natl. Acad. Sci. U.S.A. 88, 1621–1625. 10.1073/pnas.88.5.1621 2000370 PMC51076

[B23] Hein G. Knight R. T. (2008). Superior temporal sulcus–It's my area: or is it? J. Cogn. Neurosci. 20, 2125–2136. 10.1162/jocn.2008.20148 18457502

[B24] Huk A. C. Dougherty R. F. Heeger D. J. (2002). Retinotopy and functional subdivision of human areas MT and MST. J. Neurosci. 22, 7195–7205. 12177214 10.1523/JNEUROSCI.22-16-07195.2002 PMC6757870

[B25] Ishai A. Ungerleider L. G. Martin A. Haxby J. V. (2000). The representation of objects in the human occipital and temporal cortex. J. Cogn. Neurosci. 12(Suppl. 2), 35–51. 10.1162/089892900564055 11506646

[B26] Kavcic V. Vaughn W. Duffy C. J. (2011). Distinct visual motion processing impairments in aging and Alzheimer's disease. Vision Res. 51, 386–395. 10.1016/j.visres.2010.12.004 21156185 PMC3061234

[B27] Kim N. G. Park J. H. (2010). Learning to perceive structure from motion and neural plasticity in patients with Alzheimer's disease. Neuropsychologia 48, 1464–1471. 10.1016/j.neuropsychologia.2010.01.016 20116388

[B28] Kirby E. Bandelow S. Hogervorst E. (2010). Visual impairment in Alzheimer's disease: a critical review. J. Alzheimers. Dis. 21, 15–34. 10.3233/JAD-2010-080785 20182034

[B29] Kravitz D. J. Saleem K. S. Baker C. I. Mishkin M. (2011). A new neural framework for visuospatial processing. Nat. Rev. Neurosci. 12, 217–230. 10.1038/nrn3008 21415848 PMC3388718

[B30] Kravitz D. J. Saleem K. S. Baker C. I. Ungerleider L. G. Mishkin M. (2013). The ventral visual pathway: an expanded neural framework for the processing of object quality. Trends Cogn. Sci. 17, 26–49. 10.1016/j.tics.2012.10.011 23265839 PMC3532569

[B31] Kurylo D. D. Corkin S. Rizzo J. F. III Growdon J. H. (1996). Greater relative impairment of object recognition than of visuospatial abilities in Alzheimer's disease. Neuropsychology 10, 74–81. 10.1037/0894-4105.10.1.74

[B32] Laird A. R. Fox P. M. Price C. J. Glahn D. C. Uecker A. M. Lancaster J. L.. (2005). ALE meta-analysis: controlling the false discovery rate and performing statistical contrasts. Hum. Brain Mapp. 25, 155–164. 10.1002/hbm.20136 15846811 PMC6871747

[B33] Lancaster J. L. Tordesillas-Gutierrez D. Martinez M. Salinas F. Evans A. Zilles K.. (2007). Bias between MNI and Talairach coordinates analyzed using the ICBM-152 brain template. Hum. Brain Mapp. 28, 1194–1205. 10.1002/hbm.20345 17266101 PMC6871323

[B34] Liu C. Zhang W. T. Tang Y. Y. Mai X. Q. Chen H. C. Tardif T.. (2008). The Visual Word Form Area: evidence from an fMRI study of implicit processing of Chinese characters. Neuroimage 40, 1350–1361. 10.1016/j.neuroimage.2007.10.014 18272399

[B35] Mandal P. K. Joshi J. Saharan S. (2012). Visuospatial perception: an emerging biomarker for Alzheimer's disease. J. Alzheimers. Dis. 31(Suppl. 3), S117–S135. 10.3233/JAD-2012-120901 22810101

[B36] Mano Q. R. Humphries C. Desai R. H. Seidenberg M. S. Osmon D. C. Stengel B. C.. (2013). The role of left occipitotemporal cortex in reading: reconciling stimulus, task, and lexicality effects. Cereb. Cortex 23, 988–1001. 10.1093/cercor/bhs093 22505661 PMC3593581

[B37] Morrone M. C. Tosetti M. Montanaro D. Fiorentini A. Cioni G. Burr D. C. (2000). A cortical area that responds specifically to optic flow, revealed by fMRI. Nat. Neurosci. 3, 1322–1328. 10.1038/81860 11100154

[B38] Pihlajamaki M. Tanila H. Kononen M. Hanninen T. Aronen H. J. Soininen H. (2005). Distinct and overlapping fMRI activation networks for processing of novel identities and locations of objects. Eur. J. Neurosci. 22, 2095–2105. 10.1111/j.1460-9568.2005.04380.x 16262647

[B39] Reisberg D. (2010). Cognition, 4th Edn. New York, NY: W. W. Norton & Company, Inc.

[B40] Rizzo M. Anderson S. W. Dawson J. Nawrot M. (2000). Vision and cognition in Alzheimer's disease. Neuropsychologia 38, 1157–1169. 10.1016/S0028-3932(00)00023-3 10838150

[B41] Rodrigue K. M. Kennedy K. M. Park D. C. (2009). Beta-amyloid deposition and the aging brain. Neuropsychol. Rev. 19, 436–450. 10.1007/s11065-009-9118-x 19908146 PMC2844114

[B42] Sakai K. L. (2005). Language acquisition and brain development. Science 310, 815–819. 10.1126/science.1113530 16272114

[B43] Sarubbo S. De Benedictis A. Maldonado I. L. Basso G. Duffau H. (2013). Frontal terminations for the inferior fronto-occipital fascicle: anatomical dissection, DTI study and functional considerations on a multi-component bundle. Brain Struct. Funct. 218, 21–37. 10.1007/s00429-011-0372-3 22200882

[B44] Schenk T. McIntosh R. D. (2010). Do we have independent visual streams for perception and action? Cogn. Neurosci. 1, 52–62. 10.1080/17588920903388950 24168245

[B45] Sereno M. I. Dale A. M. Reppas J. B. Kwong K. K. Belliveau J. W. Brady T. J.. (1995). Borders of multiple visual areas in humans revealed by functional magnetic resonance imaging. Science 268, 889–893. 10.1126/science.7754376 7754376

[B46] Silveri M. C. Leggio M. G. (1996). Influence of disorders of visual perception in word-to-picture matching tasks in patients with Alzheimer's disease. Brain Lang. 54, 326–334. 10.1006/brln.1996.0078 8811963

[B47] Talairach J. Tournoux P. (1988). Co-planar Stereotaxic Atlas of the Human Brain: 3-Dimensional Proportional System: An Approach to Medical Cerebral Imaging, New York, NY: Thieme.

[B48] Tootell R. B. Mendola J. D. Hadjikhani N. K. Ledden P. J. Liu A. K. Reppas J. B.. (1997). Functional analysis of V3A and related areas in human visual cortex. J. Neurosci. 17, 7060–7078. 9278542 10.1523/JNEUROSCI.17-18-07060.1997 PMC6573277

[B49] Turkeltaub P. E. Eden G. F. Jones K. M. Zeffiro T. A. (2002). Meta-analysis of the functional neuroanatomy of single-word reading: method and validation. Neuroimage 16, 765–780. 10.1006/nimg.2002.1131 12169260

[B50] Turkeltaub P. E. Eickhoff S. B. Laird A. R. Fox M. Wiener M. Fox P. (2012). Minimizing within-experiment and within-group effects in Activation Likelihood Estimation meta-analyses. Hum. Brain Mapp. 33, 1–13. 10.1002/hbm.21186 21305667 PMC4791073

[B51] Tyler C. W. Likova L. T. Chen C. C. Kontsevich L. L. Schira M. M. Wade A. R. (2005). Extended concepts of occipital retinotopy. Curr. Med. Imag. Rev 1, 319–329. 10.2174/157340505774574772

[B52] Vigneau M. Beaucousin V. Herve P. Y. Duffau H. Crivello F. Houde O.. (2006). Meta-analyzing left hemisphere language areas: phonology, semantics, and sentence processing. Neuroimage 30, 1414–1432. 10.1016/j.neuroimage.2005.11.002 16413796

[B53] Wandell B. A. Brewer A. A. Dougherty R. F. (2005). Visual field map clusters in human cortex. Philos. Trans. R. Soc. Lond. B. Biol. Sci. 360, 693–707. 10.1098/rstb.2005.1628 15937008 PMC1569486

[B54] Wandell B. A. Dumoulin S. O. Brewer A. A. (2007). Visual field maps in human cortex. Neuron 56, 366–383. 10.1016/j.neuron.2007.10.012 17964252

[B55] Weiss P. H. Marshall J. C. Zilles K. Fink G. R. (2003). Are action and perception in near and far space additive or interactive factors? Neuroimage 18, 837–846. 10.1016/S1053-8119(03)00018-1 12725760

[B56] Yovel G. Kanwisher N. (2004). Face perception: domain specific, not process specific. Neuron 44, 889–898. 10.1016/j.neuron.2004.11.018 15572118

