# Supplementary material for: Mapping the “What” and “Where” Visual Cortices and Their Atrophy in Alzheimer's Disease: Combined Activation Likelihood Estimation with Voxel-Based Morphometry
Source: Front Hum Neurosci. 2016 Jun 29;10:333. doi: 10.3389/fnhum.2016.00333 (PMC4925679; doi:10.3389/fnhum.2016.00333)
Supplement: Supplementary file 1 [file Table1.DOCX]

***Supplementary Material***

**Mapping the “What” and “Where” Visual Cortices and Their Atrophy in Alzheimer’s Disease: Combined Activation Likelihood Estimation With Voxel-based Morphometry**

Yan-Jia Deng, Lin Shi ^#^, Yi Lei, Peipeng Liang, Kuncheng Li, Winnie CW Chu, Defeng Wang ^##^, for the Alzheimer’s Disease Neuroimaging Initiative*

^#^ Corresponding author: Lin Shi; Email: [shilin@cuhk.edu.hk](mailto:shilin@cuhk.edu.hk)

^##^ Corresponding author: Defeng Wang; Email: [dfwang@cuhk.edu.hk](mailto:dfwang@cuhk.edu.hk)

**Introduction of ADNI dataset**

The ADNI was launched in 2003 as a public-private partnership, led by Principal Investigator Michael W. Weiner, MD. The primary goal of ADNI has been to test whether serial MRI, positron emission tomography (PET), other biological markers, and clinical and neuropsychological assessment can be combined to measure the progression of MCI and AD.

ADNI is funded by the National Institute on Aging, the National Institute of Biomedical Imaging and Bioengineering, and through generous contributions from the following: AbbVie, Alzheimer’s Association; Alzheimer’s Drug Discovery Foundation; Araclon Biotech; BioClinica, Inc.; Biogen; Bristol-Myers Squibb Company; CereSpir, Inc.; Eisai Inc.; Elan Pharmaceuticals, Inc.; Eli Lilly and Company; EuroImmun; F. Hoffmann-La Roche Ltd and its affiliated company Genentech, Inc.; Fujirebio; GE Healthcare; IXICO Ltd.; Janssen Alzheimer Immunotherapy Research & Development, LLC.; Johnson & Johnson Pharmaceutical

Research & Development LLC.; Lumosity; Lundbeck; Merck & Co., Inc.; Meso Scale Diagnostics, LLC.; NeuroRx Research; Neurotrack Technologies; Novartis Pharmaceuticals Corporation; Pfizer Inc.; Piramal Imaging; Servier; Takeda Pharmaceutical Company; and Transition Therapeutics. The Canadian Institutes of Health Research is providing funds to support ADNI clinical sites in Canada. Private sector contributions are facilitated by the Foundation for the National Institutes of Health (www.fnih.org). The grantee organization is the Northern California Institute for Research and Education, and the study is coordinated by the Alzheimer's Disease Cooperative Study at the University of California, San Diego. ADNI data are disseminated by the Laboratory for Neuro Imaging at the University of Southern California.

**Supplementary Tables**

Supplementary Table 1. Summary of studies selected for the meta-analysis

| Category/study(author) | Year | No. of Subjects | Contrast | No. of Foci |
| --- | --- | --- | --- | --- |
| ***Face vision*** |  |  |  |  |
| Leube et al. (2003) | 2003 | 13 | Face > baseline | 5 |
| Badzakova-Trajkov et al. (2010) | 2010 | 155 | Face > baseline | 18 |
| Love et al. (2011) | 2011 | 20 | Face > baseline | 10 |
| Vuilleumier et al. (2003) | 2003 | 13 | Face > baseline | 11 |
| Henson et al. (2003) | 2003 | 21 | Face > scrambled image | 4 |
| Furl et al. (2007) | 2007 | 12 | Face > baseline | 7 |
| Fruhholz et al. (2011) | 2011 | 24 | Face > scrambled image | 21 |
| Platek et al. (2009) | 2009 | 12 | Face > baseline | 1 |
| Schwartz et al. (2013) | 2013 | 8 | Face > baseline | 3 |
| Joseph et al. (2011) | 2011 | 43 | Face > baseline | 11 |
| Jehna et al. (2011) | 2011 | 30 | Face > scrambled image | 5 |
| Benuzzi et al. (2007) | 2007 | 24 | Face > noise | 36 |
| Rossion et al. (2003) | 2003 | 11 | Face > baseline | 4 |
| Rossion et al. (2012) | 2012 | 40 | Face > scrambled image | 12 |
| Gobbini et al. (2011) | 2011 | 12 | Face > baseline | 30 |
| Hoffman et al. (2000) | 2000 | 9 | Face > baseline | 8 |
| Pagliaccio et al. (2013) | 2013 | 52 | Face > baseline | 1 |
| Ramon et al. (2010) | 2010 | 13 | Face > scrambled image | 13 |
| Blonder et al. (2004) | 2004 | 14 | Face > baseline | 6 |
| Liu et al. (2009) | 2009 | 17 | Face > baseline | 11 |
| Orlov et al. (2010) | 2010 | 12 | Face > baseline | 10 |
| Jiang et al. (2015) | 2015 | 12 | Face > scrambled image | 6 |
| Pegors et al. (2015) | 2015 | 28 | Face > baseline | 3 |
| Matsuyoshi et al. (2015) | 2015 | 20 | Face > scrambled image | 28 |
| Soria et al. (2015) | 2015 | 15 | Face > fixation | 51 |
| Hermann (2015) | 2015 | 26 | Face > noise | 2 |
| Freeman et al. (2015) | 2015 | 31 | Face > baseline | 1 |
| ***Word/ letter vision*** |  |  |  |  |
| Olulade et al. (2013) | 2013 | 15 | Word > baseline | 8 |
| Joseph et al. (2006) | 2006 | 11 | Word > baseline | 7 |
| Minati et al. (2008) | 2008 | 10 | Word > baseline | 15 |
| Szwed et al. (2011) | 2011 | 16 | Word > scrambled image | 9 |
| Longcamp et al. (2011) | 2011 | 10 | Word > baseline | 1 |
| Pegado et al. (2011) | 2011 | 14 | Letter > baseline | 4 |
| Dehaene et al. (2001) | 2001 | 15 | Word > noise | 6 |
| Dehaene et al. (2004) | 2004 | 26 | Word > noise | 6 |
| Casarotto et al. (2006) | 2006 | 8 | Word > baseline | 4 |
| Peng et al. (2003) | 2003 | 8 | Word > baseline | 34 |
| Gold et al. (2007) | 2007 | 16 | Word > baseline | 2 |
| Gros et al. (2001) | 2001 | 6 | Letter > baseline | 2 |
| James et al. (2006) | 2009 | 19 | Letter > baseline | 12 |
| Glezer et al. (2015) | 2015 | 12 | Word > scrambled image | 1 |
| Muayqil et al. (2015) | 2015 | 14 | Word > scramble image | 4 |
| Cavian-Pratesi et al. (2015) | 2015 | 11 | Word > scramble image | 4 |
| ***Object vision*** |  |  |  |  |
| Thoma et al. (2011) | 2011 | 17 | Object > scrambled image | 8 |
| Pegado et al. (2011) | 2011 | 14 | Object > baseline | 6 |
| Yamamoto et al. (2008) | 2008 | 6 | Object > baseline | 3 |
| Podzebenko et al. (2005) | 2005 | 16 | Object > baseline | 13 |
| Minati et al. (2008) | 2008 | 10 | Object > baseline | 23 |
| Szwed et al. (2011) | 2011 | 16 | Object > scrambled image | 7 |
| Creem-Regehr et al. (2007) | 2007 | 23 | Object > scrambled image | 10 |
| Durand et al. (2009) | 2009 | 17 | Object > scrambled image | 6 |
| Cardin et al. (2011) | 2011 | 14 | Object > baseline | 9 |
| Valyear et al. (2006) | 2006 | 7 | Object > baseline | 1 |
| Niemeier et al. (2005) | 2005 | 10 | Object > baseline | 2 |
| Casarotto et al. (2006) | 2006 | 8 | Object > baseline | 1 |
| Peelen et al. (2012) | 2012 | 26 | Object > baseline | 2 |
| Bucher et al. (2006) | 2006 | 16 | Object > baseline | 7 |
| Blonder et al. (2004) | 2004 | 14 | Object > baseline | 6 |
| Karanian (2015) | 2015 | 14 | Object > scrambled image | 4 |
| Nishimura et al. (2015) | 2015 | 16 | Object > scrambled image | 2 |
| Yu et al. (2015) | 2015 | 7 | Object > scrambled image | 2 |
| ***Motion vision*** |  |  |  |  |
| Homola et al. (2012) | 2012 | 24 | Motion > baseline | 2 |
| Schraa-Tam et al. (2008) | 2008 | 22 | Motion > baseline | 12 |
| Morito, Tanabe et al. (2009) | 2009 | 28 | Motion > baseline | 17 |
| Caplan et al. (2006) | 2006 | 12 | Motion > baseline | 16 |
| Katsuyama et al. (2011) | 2011 | 31 | Motion > baseline | 3 |
| Podzebenko et al. (2005) | 2005 | 16 | Motion > baseline | 9 |
| Braddick et al. (2001) | 2001 | 3 | Motion > noise | 14 |
| Braddick et al. (2000) | 2000 | 4 | Motion > baseline | 4 |
| Oreja-Guevara et al. (2004) | 2004 | 9 | Motion > baseline | 61 |
| Bucher et al. (2006) | 2006 | 16 | Motion > baseline | 12 |
| Santi et al. (2003) | 2003 | 10 | Motion > baseline | 14 |
| Aso et al. (2007) | 2007 | 12 | Motion > baseline | 5 |
| Qian et al. (2015) | 2015 | 20 | Motion > baseline | 9 |
| Jednorog et al. (2015) | 2015 | 14 | Motion > baseline | 21 |
| ***Spatial vision*** |  |  |  |  |
| Iwami et al. (2002) | 2002 | 10 | Spatial > baseline | 11 |
| Grady et al. (2014) | 2014 | 14 | Spatial > baseline | 11 |
| Joseph et al. (2003) | 2003 | 10 | Spatial > baseline | 4 |
| Joseph et al. (2003) | 2003 | 11 | Spatial > baseline | 5 |
| Joseph et al. (2003) | 2003 | 10 | Spatial > baseline | 3 |
| Joseph et al. (2003) | 2003 | 11 | Spatial > baseline | 3 |
| Zeidman et al. (2012) | 2012 | 19 | Spatial > baseline | 2 |
| Yamamoto et al. (2008) | 2008 | 13 | Spatial > baseline | 30 |
| Katsuyama et al. (2011) | 2011 | 31 | Spatial > baseline | 7 |
| Fraedrich et al. (2010) | 2010 | 18 | Spatial > baseline | 6 |
| Georgieva et al. (2008) | 2008 | 18 | Spatial > scrambled image | 12 |
| Creem-Regehr et al. (2007) | 2007 | 23 | Spatial > scrambled image | 6 |
| Kana et al. (2013) | 2013 | 14 | Spatial > baseline | 11 |
| Wu et al. (2012) | 2012 | 19 | Spatial > baseline | 9 |
| Valyear et al. (2006) | 2006 | 3 | Spatial > baseline | 1 |
| Braddick et al. (2000) | 2000 | 4 | Spatial > baseline | 8 |
| Kaufmann et al. (1997) | 2008 | 12 | Spatial > baseline | 3 |
| Sterzer et al. (2005) | 2005 | 12 | Spatial > baseline | 4 |
| Brouwer et al. (2005) | 2005 | 7 | Spatial > baseline | 7 |
| Cant et al. (2007) | 2007 | 9 | Spatial > baseline | 10 |
| Schoth et al. (2007) | 2007 | 22 | Spatial > baseline | 12 |
| Dumoulin et al. (2007) | 2004 | 4 | Spatial > noise | 2 |
| Baecke et al. (2009) | 2009 | 26 | Spatial > baseline | 6 |
| Gros et al. (2001) | 2001 | 6 | Spatial > baseline | 2 |
| Ritzl et al. (2003) | 2003 | 11 | Spatial > baseline | 10 |
| Negawa et al. (2002) | 2003 | 13 | Spatial > scrambled image | 13 |
| Aso et al. (2007) | 2007 | 12 | Spatial > baseline | 4 |
| Straube et al (2011) | 2011 | 10 | Spatial > scrambled image | 4 |
| Prvulovic et al. (2002) | 2002 | 14 | Spatial > baseline | 29 |
| Bray et al. (2015) | 2015 | 26 | Spatial > baseline | 12 |
| ***Scene vision**** |  |  |  |  |
| Amemiya et al. (2012) | 2012 | 27 | Scene > baseline | 10 |
| Jehna et al. (2011) | 2011 | 30 | Scene > baseline | 4 |
| Blonder et al. (2004) | 2004 | 14 | Scene > baseline | 10 |
| Bradley et al. (2015) | 2015 | 24 | Scene > baseline | 30 |
| ***Color vision**** |  |  |  |  |
| Claeys et al. (2004) | 2004 | 16 | Color > baseline | 10 |
| Leh et al. (2010) | 2009 | 1 | Color > baseline | 4 |
| Kaufmann et al. (2008) | 2008 | 12 | Color > baseline | 1 |
| ***Body vision**** |  |  |  |  |
| Orlov et al. (2010) | 2010 | 12 | Body > baseline | 18 |
| Soria et al. (2015) | 2015 | 15 | Body > fixation | 49 |

* Coordinates of these experiments were only included in the “what” vision to perform contrast analysis with “where” vision

Supplementary Table 2. Demographics of subjects and mini–mental state examination (MMSE) score

|  | CN | EMCI | LMCI | AD | *p* value |
| --- | --- | --- | --- | --- | --- |
| Age (years) | 73.6±5.8 | 71.2±6.9 | 70.2±7.9 | 72.5±7.5 | 0.159 |
| Gender (number of male/female) | 19/25 | 21/31 | 22/13 | 15/15 | 0.193 |
| Education (years) | 16.27±0.35 | 15.67±0.38 | 16.74±0.47 | 15.73±0.49 | 0.203 |
| MMSE score | 28.77±1.36 | 28.19±1.75 | 27.57±1.75 | 22.63±2.90 | 7.16×10^-30^ |

Statistical level: *p* < 0.05

CN: normal control; EMCI: early stage of mild cognitive impairment (MCI); LMCI: late stage of MCI; AD: Alzheimer’s disease.

**References:**

Amemiya, S. and Ohtomo, K. (2012). Effect of the observed pupil size on the amygdala of the beholders. Soc Cogn Affect Neurosci*,* 7(3), 332-341.

Aso, T., et al. (2007). Subregions of human parietal cortex selectively encoding object orientation. Neurosci Lett*,* 415(3), 225-230.

Badzakova-Trajkov, G., et al. (2010). Cerebral asymmetries: complementary and independent processes. PLoS One*,* 5(3), e9682.

Baecke, S., et al. (2009). Event-related functional magnetic resonance imaging (efMRI) of depth-by-disparity perception: additional evidence for right-hemispheric lateralization. Exp Brain Res*,* 196(3), 453-458.

Benuzzi, F., et al. (2007). Processing the socially relevant parts of faces. Brain Res Bull*,* 74(5), 344-356.

Blonder, L. X., et al. (2004). Regional brain response to faces of humans and dogs. Brain Res Cogn Brain Res*,* 20(3), 384-394.

Braddick, O. J., et al. (2000). Form and motion coherence activate independent, but not dorsal/ventral segregated, networks in the human brain. Curr Biol*,* 10(12), 731-734.

Braddick, O. J., et al. (2001). Brain areas sensitive to coherent visual motion. Perception*,* 30(1), 61-72.

Bradley, M. M., et al. (2015). Imaging distributed and massed repetitions of natural scenes: spontaneous retrieval and maintenance. Hum Brain Mapp*,* 36(4), 1381-1392.

Bray, S., et al. (2015). Intraparietal sulcus activity and functional connectivity supporting spatial working memory manipulation. Cereb Cortex*,* 25(5), 1252-1264.

Brouwer, G. J., et al. (2005). Activation in visual cortex correlates with the awareness of stereoscopic depth. J Neurosci*,* 25(45), 10403-10413.

Bucher, K., et al. (2006). Maturation of luminance- and motion-defined form perception beyond adolescence: a combined ERP and fMRI study. Neuroimage*,* 31(4), 1625-1636.

Cant, J. S. and Goodale, M. A. (2007). Attention to form or surface properties modulates different regions of human occipitotemporal cortex. Cereb Cortex*,* 17(3), 713-731.

Caplan, J. B., et al. (2006). Parallel networks operating across attentional deployment and motion processing: a multi-seed partial least squares fMRI study. Neuroimage*,* 29(4), 1192-1202.

Cardin, V., et al. (2011). Top-down modulations in the visual form pathway revealed with dynamic causal modeling. Cereb Cortex*,* 21(3), 550-562.

Casarotto, S., et al. (2006). Combination of event-related potentials and functional magnetic resonance imaging during single-letter reading. Conf Proc IEEE Eng Med Biol Soc*,* 1, 984-987.

Cavina-Pratesi, C., et al. (2015). Visual processing of words in a patient with visual form agnosia: a behavioural and fMRI study. Cortex*,* 64, 29-46.

Claeys, K. G., et al. (2004). Color discrimination involves ventral and dorsal stream visual areas. Cereb Cortex*,* 14(7), 803-822.

Creem-Regehr, S. H., et al. (2007). The influence of complex action knowledge on representations of novel graspable objects: evidence from functional magnetic resonance imaging. J Int Neuropsychol Soc*,* 13(6), 1009-1020.

Dehaene, S., et al. (2001). Cerebral mechanisms of word masking and unconscious repetition priming. Nat Neurosci*,* 4(7), 752-758.

Dehaene, S., et al. (2004). Letter binding and invariant recognition of masked words: behavioral and neuroimaging evidence. Psychol Sci*,* 15(5), 307-313.

Dumoulin, S. O. and Hess, R. F. (2007). Cortical specialization for concentric shape processing. Vision Res*,* 47(12), 1608-1613.

Durand, J. B., et al. (2009). Parietal regions processing visual 3D shape extracted from disparity. Neuroimage*,* 46(4), 1114-1126.

Fraedrich, E. M., et al. (2010). Spatiotemporal phase-scrambling increases visual cortex activity. Neuroreport*,* 21(8), 596-600.

Freeman, J. B., et al. (2015). The neural basis of contextual influences on face categorization. Cereb Cortex*,* 25(2), 415-422.

Fruhholz, S., et al. (2011). Face recognition under ambiguous visual stimulation: fMRI correlates of "encoding styles". Hum Brain Mapp*,* 32(10), 1750-1761.

Furl, N., et al. (2007). Face adaptation aftereffects reveal anterior medial temporal cortex role in high level category representation. Neuroimage*,* 37(1), 300-310.

Georgieva, S. S., et al. (2008). The extraction of 3D shape from texture and shading in the human brain. Cereb Cortex*,* 18(10), 2416-2438.

Glezer, L. S., et al. (2015). Adding words to the brain's visual dictionary: novel word learning selectively sharpens orthographic representations in the VWFA. J Neurosci*,* 35(12), 4965-4972.

Gobbini, M. I., et al. (2011). Distinct neural systems involved in agency and animacy detection. J Cogn Neurosci*,* 23(8), 1911-1920.

Grady, C. L., et al. (2014). Early visual deprivation from congenital cataracts disrupts activity and functional connectivity in the face network. Neuropsychologia*,* 57, 122-139.

Grol, M. J., et al. (2007). Parieto-frontal connectivity during visually guided grasping. J Neurosci*,* 27(44), 11877-11887.

Gros, H., et al. (2001). Event-related functional magnetic resonance imaging study of the extrastriate cortex response to a categorically ambiguous stimulus primed by letters and familiar geometric figures. J Cereb Blood Flow Metab*,* 21(11), 1330-1341.

Henson, R. N., et al. (2003). Electrophysiological and haemodynamic correlates of face perception, recognition and priming. Cereb Cortex*,* 13(7), 793-805.

Hermann, P., et al. (2015). Neural basis of identity information extraction from noisy face images. J Neurosci*,* 35(18), 7165-7173.

Hoffman, E. A. and Haxby, J. V. (2000). Distinct representations of eye gaze and identity in the distributed human neural system for face perception. Nat Neurosci*,* 3(1), 80-84.

Homola, G. A., et al. (2012). A brain network processing the age of faces. PLoS One*,* 7(11), e49451.

Iwami, T., et al. (2002). Common neural processing regions for dynamic and static stereopsis in human parieto-occipital cortices. Neurosci Lett*,* 327(1), 29-32.

James, K. H. and Gauthier, I. (2006). Letter processing automatically recruits a sensory-motor brain network. Neuropsychologia*,* 44(14), 2937-2949.

Jednorog, K., et al. (2015). Three-dimensional grammar in the brain: Dissociating the neural correlates of natural sign language and manually coded spoken language. Neuropsychologia*,* 71, 191-200.

Jehna, M., et al. (2011). The functional correlates of face perception and recognition of emotional facial expressions as evidenced by fMRI. Brain Res*,* 1393, 73-83.

Jiang, F., et al. (2015). Category search speeds up face-selective fMRI responses in a non-hierarchical cortical face network. Cortex*,* 66, 69-80.

Joseph, J. E., et al. (2006). fMRI correlates of cortical specialization and generalization for letter processing. Neuroimage*,* 32(2), 806-820.

Joseph, J. E., et al. (2011). Progressive and regressive developmental changes in neural substrates for face processing: testing specific predictions of the Interactive Specialization account. Dev Sci*,* 14(2), 227-241.

Joseph, J. E. and Gathers, A. D. (2003). Effects of structural similarity on neural substrates for object recognition. Cogn Affect Behav Neurosci*,* 3(1), 1-16.

Kana, R. K., et al. (2013). The local, global, and neural aspects of visuospatial processing in autism spectrum disorders. Neuropsychologia*,* 51(14), 2995-3003.

Kanwisher, N., et al. (1997). The fusiform face area: a module in human extrastriate cortex specialized for face perception. J Neurosci*,* 17(11), 4302-4311.

Karanian, J. M. and Slotnick, S. D. (2015). Memory for shape reactivates the lateral occipital complex. Brain Res*,* 1603, 124-132.

Katsuyama, N., et al. (2011). Perception of object motion in three-dimensional space induced by cast shadows. Neuroimage*,* 54(1), 485-494.

Kaufmann, L., et al. (2008). A developmental fMRI study of nonsymbolic numerical and spatial processing. Cortex*,* 44(4), 376-385.

Leh, S. E., et al. (2010). Blindsight mediated by an S-cone-independent collicular pathway: an fMRI study in hemispherectomized subjects. J Cogn Neurosci*,* 22(4), 670-682.

Leube, D. T., et al. (2003). Brain regions sensitive to the face inversion effect: a functional magnetic resonance imaging study in humans. Neurosci Lett*,* 342(3), 143-146.

Liu, J., et al. (2009). Similarities in neural activations of face and Chinese character discrimination. Neuroreport*,* 20(3), 273-277.

Longcamp, M., et al. (2011). What differs in visual recognition of handwritten vs. printed letters? An fMRI study. Hum Brain Mapp*,* 32(8), 1250-1259.

Love, S. A., et al. (2011). Cerebral correlates and statistical criteria of cross-modal face and voice integration. Seeing Perceiving*,* 24(4), 351-367.

Matsuyoshi, D., et al. (2015). Dissociable cortical pathways for qualitative and quantitative mechanisms in the face inversion effect. J Neurosci*,* 35(10), 4268-4279.

Minati, L., et al. (2008). Spatial correspondence between functional MRI (fMRI) activations and cortical current density maps of event-related potentials (ERP): a study with four tasks. Brain Topogr*,* 21(2), 112-127.

Morito, Y., et al. (2009). Neural representation of animacy in the early visual areas: a functional MRI study. Brain Res Bull*,* 79(5), 271-280.

Muayqil, T., et al. (2015). Representation of visual symbols in the visual word processing network. Neuropsychologia*,* 69, 232-241.

Negawa, T., et al. (2002). M pathway and areas 44 and 45 are involved in stereoscopic recognition based on binocular disparity. Jpn J Physiol*,* 52(2), 191-198.

Niemeier, M., et al. (2005). A contralateral preference in the lateral occipital area: sensory and attentional mechanisms. Cereb Cortex*,* 15(3), 325-331.

Nishimura, M., et al. (2015). Size precedes view: developmental emergence of invariant object representations in lateral occipital complex. J Cogn Neurosci*,* 27(3), 474-491.

Olulade, O. A., et al. (2013). Developmental differences for word processing in the ventral stream. Brain Lang*,* 125(2), 134-145.

Oreja-Guevara, C., et al. (2004). The role of V5 (hMT+) in visually guided hand movements: an fMRI study. Eur J Neurosci*,* 19(11), 3113-3120.

Orlov, T., et al. (2010). Topographic representation of the human body in the occipitotemporal cortex. Neuron*,* 68(3), 586-600.

Pagliaccio, D., et al. (2013). Functional brain activation to emotional and nonemotional faces in healthy children: evidence for developmentally undifferentiated amygdala function during the school-age period. Cogn Affect Behav Neurosci*,* 13(4), 771-789.

Peelen, M. V. and Caramazza, A. (2012). Conceptual object representations in human anterior temporal cortex. J Neurosci*,* 32(45), 15728-15736.

Pegado, F., et al. (2011). Breaking the symmetry: mirror discrimination for single letters but not for pictures in the Visual Word Form Area. Neuroimage*,* 55(2), 742-749.

Pegors, T. K., et al. (2015). Common and unique representations in pFC for face and place attractiveness. J Cogn Neurosci*,* 27(5), 959-973.

Peng, D. L., et al. (2003). Neural basis of the non-attentional processing of briefly presented words. Hum Brain Mapp*,* 18(3), 215-221.

Platek, S. M. and Kemp, S. M. (2009). Is family special to the brain? An event-related fMRI study of familiar, familial, and self-face recognition. Neuropsychologia*,* 47(3), 849-858.

Podzebenko, K., et al. (2005). Real and imaginary rotary motion processing: functional parcellation of the human parietal lobe revealed by fMRI. J Cogn Neurosci*,* 17(1), 24-36.

Prvulovic, D., et al. (2002). Functional imaging of visuospatial processing in Alzheimer's disease. Neuroimage*,* 17(3), 1403-1414.

Qian, Y., et al. (2015). Magnocellular-dorsal pathway function is associated with orthographic but not phonological skill: fMRI evidence from skilled Chinese readers. Neuropsychologia*,* 71, 84-90.

Ramon, M., et al. (2010). Personally familiar faces are perceived categorically in face-selective regions other than the fusiform face area. Eur J Neurosci*,* 32(9), 1587-1598.

Ritzl, A., et al. (2003). Functional anatomy and differential time courses of neural processing for explicit, inferred, and illusory contours. An event-related fMRI study. Neuroimage*,* 19(4), 1567-1577.

Rossion, B., et al. (2003). A network of occipito-temporal face-sensitive areas besides the right middle fusiform gyrus is necessary for normal face processing. Brain*,* 126(Pt 11), 2381-2395.

Rossion, B., et al. (2012). Defining face perception areas in the human brain: a large-scale factorial fMRI face localizer analysis. Brain Cogn*,* 79(2), 138-157.

Santi, A., et al. (2003). Perceiving biological motion: dissociating visible speech from walking. J Cogn Neurosci*,* 15(6), 800-809.

Schoth, F., et al. (2007). Cerebral processing of spontaneous reversals of the rotating Necker cube. Neuroreport*,* 18(13), 1335-1338.

Schraa-Tam, C. K., et al. (2008). An fMRI study on smooth pursuit and fixation suppression of the optokinetic reflex using similar visual stimulation. Exp Brain Res*,* 185(4), 535-544.

Schwartz, B. L., et al. (2013). Neural basis of implicit memory for socio-emotional information in schizophrenia. Psychiatry Res*,* 206(2-3), 173-180.

Soria, B. D. and Suchan, B. (2015). Is the whole the sum of its parts? Configural processing of headless bodies in the right fusiform gyrus. Behav Brain Res*,* 281, 102-110.

Sterzer, P. and Kleinschmidt, A. (2005). A neural signature of colour and luminance correspondence in bistable apparent motion. Eur J Neurosci*,* 21(11), 3097-3106.

Straube, S. and Fahle, M. (2011). Visual detection and identification are not the same: evidence from psychophysics and fMRI. Brain Cogn*,* 75(1), 29-38.

Szwed, M., et al. (2011). Specialization for written words over objects in the visual cortex. Neuroimage*,* 56(1), 330-344.

Thoma, V. and Henson, R. N. (2011). Object representations in ventral and dorsal visual streams: fMRI repetition effects depend on attention and part-whole configuration. Neuroimage*,* 57(2), 513-525.

Valyear, K. F., et al. (2006). A double dissociation between sensitivity to changes in object identity and object orientation in the ventral and dorsal visual streams: a human fMRI study. Neuropsychologia*,* 44(2), 218-228.

Vuilleumier, P., et al. (2003). Distinct spatial frequency sensitivities for processing faces and emotional expressions. Nat Neurosci*,* 6(6), 624-631.

Wu, X., et al. (2012). The neural basis of impossible figures: evidence from an fMRI study of the two-pronged trident. Neurosci Lett*,* 508(1), 17-21.

Yamamoto, T., et al. (2008). Neural correlates of the stereokinetic effect revealed by functional magnetic resonance imaging. J Vis*,* 8(10), 11-14.

Yu, D., et al. (2015). Locating the cortical bottleneck for slow reading in peripheral vision. J Vis*,* 15(11), 3.

Zeidman, P., et al. (2012). Exploring the parahippocampal cortex response to high and low spatial frequency spaces. Neuroreport*,* 23(8), 503-507.
